# Diverse electron carriers drive syntrophic interactions in an enriched anaerobic acetate-oxidizing consortium

**DOI:** 10.1038/s41396-023-01542-6

**Published:** 2023-10-25

**Authors:** Elizabeth A. McDaniel, Matthew Scarborough, Daniel Girma Mulat, Xuan Lin, Pranav S. Sampara, Heather M. Olson, Robert P. Young, Elizabeth K. Eder, Isaac K. Attah, Lye Meng Markillie, David W. Hoyt, Mary S. Lipton, Steven J. Hallam, Ryan M. Ziels

**Affiliations:** 1 Department of Civil Engineering, The University of British Columbia, Vancouver, BC Canada; 2 Department of Microbiology and Immunology, The University of British Columbia, Vancouver, BC Canada; 3 Department of Civil and Environmental Engineering, University of Vermont, Burlington, VT USA; 4 Environmental and Biological Sciences Directorate, Pacific Northwest National Laboratory, Richland, WA USA; 5 ECOSCOPE Training Program, The University of British Columbia, Vancouver, BC Canada; 6 Graduate Program in Bioinformatics, The University of British Columbia, Vancouver, BC Canada; 7 Genome Science and Technology Program, The University of British Columbia, Vancouver, BC Canada; 8 Life Sciences Institute, The University of British Columbia, Vancouver, BC Canada

**Keywords:** Microbial ecology, Water microbiology

## Abstract

In many anoxic environments, syntrophic acetate oxidation (SAO) is a key pathway mediating the conversion of acetate into methane through obligate cross-feeding interactions between SAO bacteria (SAOB) and methanogenic archaea. The SAO pathway is particularly important in engineered environments such as anaerobic digestion (AD) systems operating at thermophilic temperatures and/or with high ammonia. Despite the widespread importance of SAOB to the stability of the AD process, little is known about their in situ physiologies due to typically low biomass yields and resistance to isolation. Here, we performed a long-term (300-day) continuous enrichment of a thermophilic (55 °C) SAO community from a municipal AD system using acetate as the sole carbon source. Over 80% of the enriched bioreactor metagenome belonged to a three-member consortium, including an acetate-oxidizing bacterium affiliated with DTU068 encoding for carbon dioxide, hydrogen, and formate production, along with two methanogenic archaea affiliated with *Methanothermobacter_A*. Stable isotope probing was coupled with metaproteogenomics to quantify carbon flux into each community member during acetate conversion and inform metabolic reconstruction and genome-scale modeling. This effort revealed that the two *Methanothermobacter_A* species differed in their preferred electron donors, with one possessing the ability to grow on formate and the other only consuming hydrogen. A thermodynamic analysis suggested that the presence of the formate-consuming methanogen broadened the environmental conditions where ATP production from SAO was favorable. Collectively, these results highlight how flexibility in electron partitioning during SAO likely governs community structure and fitness through thermodynamic-driven mutualism, shedding valuable insights into the metabolic underpinnings of this key functional group within methanogenic ecosystems.

## Introduction

Anaerobic digestion (AD) is a globally important biotechnology for resource recovery and biogas production from organic waste streams. As an open fermentation process, conversion of complex organic polymers into methane within AD systems depends on coordinated activities of multiple microbial trophic guilds, including hydrolyzers, fermenters, syntrophs, and methanogenic archaea [1]. In the terminal steps of the AD food-web, acetate accounts for around 70% of the total electron flow into methane [2], and its turnover rate can have significant impacts on the AD loading capacity and process stability [3].

Acetate can be converted into methane and carbon dioxide via acetoclastic methanogenesis by archaea belonging to the genera *Methanothrix* and *Methanosarcina* [4]. Alternatively, acetate can be oxidized by syntrophic acetate oxidizing (SAO) bacteria into carbon dioxide, hydrogen and/or formate, which are substrates for methanogenesis via carbon dioxide reduction by archaeal partners [5]. SAO is not thermodynamically feasible under standard conditions, and therefore it requires the presence of methanogenic archaea to maintain low hydrogen and/or formate levels [6]. Although acetoclastic and hydrogenotrophic methanogens coexist in many AD environments, oftentimes acetoclastic methanogens are rare or absent in thermophilic systems (>50 °C) and/or in systems with elevated free ammonia (NH_3_) concentrations [7]. In such AD systems, SAO is likely an important pathway for methane production [8–10].

Despite their importance in driving methane production within many anoxic environments, little is currently known about the in situ physiologies and activities of SAO bacteria (SAOB). To date, only six strains of SAOB are available in pure culture: the thermophilic *Pseudothermotoga lettingae* [11] and *Thermacetogenium phaeum* [12]; the thermotolerant *Tepidanaerobacter acetatoxydans* [13]; the mesophilic *Clostridium ultunense* [14] and *Syntrophaceticus schinkii* [15]; and the alkaliphilic *Candidatus* Contubernalis alkalaceticum [16]. All of these isolated SAOB were obtained by enrichment or co-cultivation with methanogens, except for *Candidatus* Contubernalis alkalaceticum that was enriched along with sulfate reducing bacteria. Among these characterized SAOB, three species utilize the reverse Wood–Ljungdahl (acetyl-coA) pathway for acetate oxidation (*Thermacetogenium phaeum*, *Syntrophaceticus schinkii*, *and Tepidanaerobacter acetatoxydans*) [17–19], indicating the potential for metabolic diversity within this functional guild. Correspondingly, culture-independent molecular approaches, such as small subunit ribosomal RNA (SSU, or 16S rRNA) gene amplicon sequencing and lineage or gene-specific PCR, have been used to infer the identity and dynamics of putative SAOB in AD systems [20–22]. Results have indicated that SAOB belong to phylogenetically diverse, and in most cases uncharacterized, groups [23]. While genome-resolved metagenomic efforts have recovered genomes for putative SAOB within AD systems [24, 25], the reversibility of the Wood–Ljungdahl pathway used by some SAOB [19] further obfuscates the discrimination of SAOB from homoacetogens. This knowledge gap currently hinders our ability to develop and validate appropriate ecosystem-level models for carbon flow within SAO-dominated ecosystems, which are important for informing engineering strategies to enhance AD process stability and carbon conversion efficiency.

Stable isotope probing (SIP) is a powerful molecular approach to link genomic identity with metabolic function by detecting isotope incorporation into biomolecules during growth [26]. Given its resolving power, SIP could be a useful approach to discern the in situ ecophysiology of uncultivated SAOB. While DNA/RNA-based SIP has been applied to identify potential SAOB in AD systems [27–29], these efforts have so-far relied on PCR based gene sequencing, limiting new insights into the metabolic underpinnings of SAO. In a recent study, Mosbæk et al. [30] combined SIP with metaproteomics and metagenomics to identify genomes of SAOB associated with [^13^C]-labeled peptides within full-scale AD systems. However, the low abundance of the putative SAOB, likely due to their low energy yields [6], limited the number of ^13^C-labeled peptides identified within the metaproteome [30]. We posit that such genome-resolved SIP efforts could benefit from AD biomass that is enriched in SAOB for improved recovery of molecular information.

In this study, we carried out long-term enrichments of syntrophic acetate-oxidizing consortia from a thermophilic wastewater treatment plant AD system, followed by genome-resolved metaproteogenomic SIP to track carbon flow into individual populations. Genome-resolved metagenomic annotations and measurements of carbon flux into proteins were used to inform a community-scale metabolic model, which was utilized to investigate the impact of different electron shuttles on the fitness and feasibility of SAO metabolism. The results of this study shed new insights into the role of interspecies electron transfer in SAO community metabolism and composition, while highlighting how SIP-based multi-omic approaches can be used to inform community-scale models of cryptic or uncharacterized metabolisms within complex microbiomes.

## Materials and methods

### Anaerobic enrichment chemostat set up and operation

Duplicate sterile 5 l glass continuous stirred-tank bioreactors (R1 and R2) were pre-flushed with 80:20 N_2_:CO_2_, and were inoculated with 800 ml of sludge collected from a thermophilic (55 °C) anaerobic digester at a nearby municipal wastewater treatment plant (Vancouver, Canada: 1.7 g NH_4_^+^-N/L, 185 mg NH_3_/L, pH 7.5; see Supplementary Methods for calculations). The inoculum was diluted with 2400 ml of sterile anoxic basal medium prepared as described by Plugge [31] (see Supplementary Methods for details), with the total ammonium-nitrogen (TAN) concentration adjusted to 1.0 g NH_4_^+^-N/l. The bioreactors were maintained at 55 °C by an electric heating jacket, and stirred at 100 rpm by a mechanical mixer (Applikon Biosciences). The bioreactors were fed (160 ml/day) with the anoxic and sterile bicarbonate-buffered basal medium described above, with sodium acetate (75 mM) amended as the predominant carbon source. A liquid volume of 3.2 l was maintained in the bioreactors, providing a solids retention time (SRT) and hydraulic retention time (HRT) of 20 days to mimic that of the full-scale digester used as inoculum. On day 126, the SRT/HRT was increased to 30 days. The bioreactors were operated for a total of 300 days.

The volume of biogas and methane concentration were recorded in real-time by a gas meter (BlueVCount; BlueSens GmbH, Germany) and optical infrared sensor (BCP-CH4 sensor; BlueSens GmbH, Germany), respectively. The gas meter contained a one-way check valve that prevented intrusion of air into the bioreactors. The pH and temperature were measured in real-time with an in situ pH probe (InPro3250 pH; Mettler Toledo, USA). Liquid samples were periodically collected from both bioreactors to determine TAN, volatile fatty acids (VFAs), chemical oxygen demand (COD), total solids (TS) and volatile solid (VS) (Supplementary Methods).

### Batch microcosms for stable isotope probing

After 300 days of chemostat operation, batch microcosms were established in 40 ml glass serum bottles flushed with 80:20 N_2_:CO_2_ by anoxically transferring 18 ml of digestate from a single bioreactor (R2) and sealing with butyl rubber septa. Four different incubation conditions were established in triplicate: (1) blank control (e.g., no amendment); (2) 50 mM [^12^C]-acetate; (3) 50 mM [2-^13^C]-acetate (e.g., methyl-labeled); (4) 50 mM [1,2-^13^C]-acetate (universally labeled). Acetate was added to the microcosms (2 ml) as anoxic sterilized basal medium containing [^12^C], [2-^13^C], or [1,2-^13^C] sodium acetate (isotope purity >98%, Cambridge Isotopes). Bottles were held at 55 °C in a shaking incubator at 100 rpm. Twelve replicate bottles were established for all universally-labeled acetate-amended microcosms, allowing for three triplicate sets to be sacrificed for protein extraction at 24, 144, and 408 h, and a single triplicate set for liquid sampling throughout for VFA analysis. Biomass was pelleted from 10 ml liquid samples via centrifugation (10,000 × *g*) and stored at −20 °C until protein extraction. The supernatant was filtered with 0.2 µm Titan PTFE syringe filters (Thermo Scientific) and stored at −20 °C until metabolomics analysis on NMR (see Supplementary Methods). Gas production, gas composition, carbon isotope ratios of CO_2_ and CH_4_, and VFAs were measured approximately every 3 days (Supplementary Methods). Headspace gas samples (0.5 ml) were also collected into Exetainer vials (12 ml, Labco, UK) pre-purged with N_2_ gas for subsequent analysis on isotope ratio mass spectrometry (IRMS) (Supplementary Methods).

### Metagenomic sequencing, assembly, and binning

Samples (10 ml) from both bioreactors on operational days 0, 19, 54, 81, 111, 234, and 283 were collected for short-read metagenomic sequencing (Supplementary Table S1). DNA was extracted from these samples using the FastDNA Spin Kit For Soil (MP Biomedicals, California) with minor modifications [32]. These DNA samples were fragmented and ligated with adapters using the Nextera XT DNA Library Preparation Kit (Illumina, USA) and sequenced on a NextSeq550 System (Illumina) in 2 × 150 bp paired-end mode, generating an average of 18 ± 5 Gbp per sample (Supplementary Table S1). Additionally, a sample collected on the day of the stable-isotope probing experiment (day 300) was extracted using the DNA PowerSoil Pro MagAttract kit (Qiagen) and prepared for Nanopore sequencing using the Q20^+^ Ligation Sequencing Kit (SQK-Q20EA) on a R10.4 MinION flow cell.

Adapter-removal and quality trimming was performed on short-read Illumina metagenomic samples using BBMap v38.36 [33]. For read-based taxonomic profiling, metagenome k-mer signatures were generated with sourmash (v.4.8.3) [34, 35] using the “sketch dna” command (*k* = 31), and the resulting signatures were classified against the GTDB (release 214) [36] using the sourmash “gather” command. The output was summarized at taxonomic ranks using the sourmash “tax” command and the GTDB reference sheet (https://osf.io/wxf9z/).

Each quality-filtered short-read metagenome was individually assembled into contigs using SPAdes v3.15.4 in “metagenomic” mode [37]. Reads from each sample were reciprocally mapped to each assembly using bowtie2 v2.4.4 [38]. Binning was performed on each individual assembly with metaBAT2 v2.14 [39] using differential coverage from the mapping of all samples, and bins were de-replicated using dRep v3.2.2 [40]. Bins were assessed for completeness and contamination based on the presence of conserved single copy-core genes with CheckM v1.1.3 [41].

Nanopore long-reads were basecalled using guppy v6.0.1, yielding 10.8 Gbp of passed reads. Adapters were then trimmed using porechop v0.3.2. Long-reads were then assembled using flye v2.9 with “--nano-hq” and “--meta” settings [42]. Contigs were then polished three times using Nanopore raw reads with Racon v1.4.3 [43], followed by three rounds of polishing with medaka v1.5. Additional rounds of polishing were performed with Racon v1.4.3 and polypolish v0.5.0 using Illumina short-reads from day 283. All short-read samples were then mapped to the long-read assembly to obtain differential coverage profiles using bowtie2 v2.4.4. Archaeal and bacterial single-copy core genes were identified on all contigs using Anvi’o v.7.0 [44]. The long-read contigs, differential coverage information, contig classifications, and single copy-core gene locations were imported into R for manual binning with the mmgenome2 v2.2.1 package [45].

A final set of metagenome-assembled genomes (MAGs) (Supplementary Data 1) was obtained by de-replicating all bins across the long-read and short-read assembly sets. For genomes that shared similarity above 95% ANI, the highest quality representative MAG was chosen based on completion and contamination statistics followed by genome contiguity. Taxonomic classifications of the final set of bins were assigned using the GTDB-tk v1.7.0 and release202 database [36, 46]. Relative abundance of the dereplicated set of bins in each sample was assessed by mapping metagenomic reads to the concatenated set of bins with Bowtie2 v2.4.4 and using the “relative_abundance” method in CoverM v0.6.1. Average nucleotide identity (ANI) values for genomes of interest were calculated using FastANI [47]. Proteins were predicted with Prodigal v2.6.3 [48] and functional annotations made with KofamKOALA v1.3.0 with KEGG release 103.0 [49] and MetaPathways v2.0 [50, 51]. To predict whether annotated hydrogenases and formate dehydrogenases were electron-bifurcating, we analyzed their beta subunits for the signature amino-acid motifs identified by Losey et al. [52] (Supplementary Methods). Annotated hydrogenases were also queried with HydDB [53] to determine their functions and group classifications.

### Metaproteomics sample preparation and data acquisition

Protein from cell pellet samples (200 µl) were extracted by bead beating in 100 mM ammonium bicarbonate buffer, then reduced and alkylated (Supplementary Methods). Proteins were then digested with trypsin and subsequently desalted using C18 solid phase extraction (Supplementary Methods). MS analysis was performed using 0.1 µg/µl of peptide solution injected into a Q‐Exactive HF-X mass spectrometer (Thermo Scientific), with the detailed conditions outlined in the Supplementary Methods.

### Metaproteomics data analysis

Mass spectrometry (MS) data for each biological replicate at all time points (*n* = 18) were analyzed using an implementation of OpenMS [54] implemented in KNIME [55] (see Supplementary Methods). Briefly, MS/MS spectra were searched using the MS-GF+ tool [56] against a protein database consisting of all ORFs from the de-replicated set of MAGs, concatenated with reversed (decoy) sequences of all protein entries. Peptide spectra matches (PSMs) were filtered at a 5% false discovery rate (FDR) with Percolator [57]. For label-free quantification (LFQ) of proteins, PSMs from unlabeled (^12^C) samples were used for protein inference with Fido [58], followed by protein FDR filtering at 5%. Protein quantification was based on the summed intensities of all unique PSMs within a protein group.

A “total protein approach” [59, 60] was implemented to infer absolute protein levels based on the LFQ data. The relative protein abundance was determined as:

$${{{{{{\rm{Relative}}}}}}}\,{{{{{{\rm{Protein}}}}}}}\,{{{{{{\rm{Abundance}}}}}}}_{i,{{{{{{\rm{sample}}}}}}}}\left(\frac{{{{{{\rm{g}}}}}}}{{{{{{{\rm{g}}}}}}}_{{{{{{{\rm{sample}}}}}}}}}\right)=\frac{{\left[{{{{{{\rm{LFQ}}}}}}}\,{{{{{{\rm{intensity}}}}}}}\right]}_{i,{{{{{{\rm{sample}}}}}}}}}{\mathop{\sum}\limits_{{{{{{{\rm{all}}}}}}}\,j\,{{{{{{\rm{in}}}}}}}\,K}{\left[{{{{{{\rm{LFQ}}}}}}}\,{{{{{{\rm{intensity}}}}}}}\right]}_{j}}$$

where *i* refers to a single protein and *K* refers to the set of all proteins in a given sample.

The total protein concentration was then estimated by multiplying the relative protein abundance (g/g) by the total protein concentration in the sample (g/L):

$${{{{{{\rm{Total}}}}}}}\,{{{{{{\rm{Protein}}}}}}}_{i,{{{{{{\rm{sample}}}}}}}}\left(\frac{{{{{{\rm{g}}}}}}}{{{{{{\rm{L}}}}}}}\right)= 	{\left[{{{{{{\rm{Relative}}}}}}}\,{{{{{{\rm{Protein}}}}}}}\,{{{{{{\rm{Abundance}}}}}}}\left(\frac{{{{{{\rm{g}}}}}}}{{{{{{{\rm{g}}}}}}}_{{{{{{{\rm{sample}}}}}}}}}\right)\right]}_{i,{{{{{{\rm{sample}}}}}}}}\\ 	\times {\left[{{{{{{\rm{Protein}}}}}}}\,{{{{{{\rm{con}}}}}}}\left(\frac{{{{{{\rm{g}}}}}}}{{{{{{\rm{L}}}}}}}\right)\right]}_{{{{{{{\rm{sample}}}}}}}}$$

Molar concentrations of proteins were determined for use in expression profiling as:

$${{{{{{\rm{Total}}}}}}}\,{{{{{{\rm{Protein}}}}}}}_{i,{{{{{{\rm{sample}}}}}}}}({{{{{{{\rm{nmole}}}}}}}}_{i}/{{{{{\rm{L}}}}}})=\frac{{\left[{{{{{{\rm{Total}}}}}}}\,{{{{{{\rm{Protein}}}}}}}\left(\frac{{{{{{\rm{g}}}}}}}{{{{{{\rm{L}}}}}}}\right)\right]}_{i,{{{{{{\rm{sample}}}}}}}}}{\left(M{W}_{i}\right)}\times ({10}^{9}{{{{{{\rm{nmole}}}}}}}/{{{{{{\rm{mole}}}}}}})$$

where *MW*_*i*_ is the molecular weight of protein *i* (g/mole), inferred from the amino acid sequence.

To identify labeled peptides in the MS data, we used MetaProSIP [61] implemented through OpenMS (see Supplementary Methods). The output of this analysis yielded estimates of the “labeling ratio” (LR), or the mass ratio of the labeled to the unlabeled peptide, and the “relative isotope abundance” (RIA), or atom percentage of ^13^C incorporated into the labeled peptide [61]. We combined the total protein concentrations along with their RIA and LR to estimate the total ^13^C-protein produced per MAG:

$${\left[{\,}^{13}{{{{{\rm{C}}}}}} \, {{{{{{\rm{protein}}}}}}}\right]}_{{{{{{{\rm{MAG}}}}}}}_{n},{{{{{{\rm{sample}}}}}}}}\left(\frac{{{{{{{\rm{mg}}}}}}}}{{{{{{\rm{L}}}}}}}\right)= 	\left(\mathop{\sum}\limits_{{{{{{{\rm{all}}}}}}}\,j\,{{{{{{\rm{in}}}}}}}\,{{{{{{\rm{MAG}}}}}}}_{n}}{\left[{{{{{{\rm{Total}}}}}}}\,{{{{{{\rm{Protein}}}}}}}\left(\frac{{{{{{{\rm{mg}}}}}}}}{{{{{{\rm{L}}}}}}}\right)\right]}_{j,{{{{{{\rm{sample}}}}}}}}\right)\\ 	 \times \left({\widehat{{{{{{{\rm{RIA}}}}}}}}}_{{{{{{{\rm{MAG}}}}}}}_{n},{{{{{{\rm{sample}}}}}}}}\right)\times \left({\widehat{{{{{{{\rm{LR}}}}}}}}}_{{{{{{{\rm{MAG}}}}}}}_{n},{{{{{{\rm{sample}}}}}}}}\right)$$

where *j* represents proteins within a given MAG (MAG_*n*_), $\widehat{{{{{{{\rm{RIA}}}}}}}}$ is the mean RIA of all proteins in MAG_*n*_ within a sample, and $\widehat{{{{{{{\rm{LR}}}}}}}}$ is the mean LR of all proteins in MAG_*n*_ within a sample.

### Metabolic reconstruction and metabolic modeling

Metabolic reconstruction and modeling were performed using CobraPy [62]. The central carbon and energy metabolism of the three most abundant MAGs (two methanogenic organisms and one syntrophic acetate oxidizing organism) were manually reconstructed based on genome annotations (see above). All reactions were confirmed to be balanced for charge and mass. In total, the metabolic model contained 65 reactions and 82 metabolites (Supplementary Data 2) across four compartments: intracellular space of the three guilds and the extracellular space. Parsimonious flux balance analysis (pFBA), flux variability analysis (FVA), and flux sampling were used to predict flux distributions through the metabolic networks and exchange of metabolic end products between microbial populations. Gibbs free energies were calculated for the population metabolisms based on their stoichiometries predicted by pFBA and FVA, using standard energies of formation of products and reactants and adjustment based on in situ concentrations (see Supplementary Data 2).

## Results and discussion

### Continuous enrichment of acetate-oxidizing consortia from a full-scale anaerobic digester

An acetate oxidizing microbial community was enriched from the sludge of a full-scale anaerobic digester for 300 days using two parallel continuously-operated chemostat reactors held at 55 °C and fed with anaerobic medium containing acetate as the primary carbon source. Low acetate levels in the effluent of the reactors (~2 mM) relative to the feed (75 mM) and high percentages of methane in the headspace indicated efficient methanogenic conversion of the substrate under the steady-state conditions (Table 1).

**Table 1:** Summary of operational parameters for acetate-fed anoxic chemostat enrichment reactors.

Parameter	Value
Influent acetate concentration (mM)	75
Hydraulic/solids retention time (days)	30
pH	8.0 ± 0.3
Total gas production rate (ml/l/day)	52 ± 20
Methane production rate (ml/l/day)	38 ± 18
Methane fraction of headspace (%)	76 ± 5
COD recovery as methane (%)	80 ± 38
Total solids (g/l)	8.5 ± 0.03
Total ammonium nitrogen (g NH_4_-N/l)	1.6 ± 0.1
Free ammonia (g NH_3_-N/l)	0.45 ± 0.03
Effluent acetate (mM)	2.0 ± 0.4

Values represent an average for the duplicate enrichment reactors during the period of steady-state performance (days 200–300). Uncertainty in values is represented by the standard deviation of the mean. Gas volumes are given for standard temperature and pressure.

Free ammonia is a known driver of microbial community structure in AD microbiomes [63, 64]. In particular high free ammonia levels in AD have been associated with inhibition of acetoclastic methanogenesis and a corresponding shift toward SAO [9, 65]. In mesophilic AD communities, the reported critical free ammonia level for this shift is around 140 to 280 mg NH_3_-N/l [65], while in thermophilic AD systems this shift toward SAO has been observed at 200 to 500 mg NH_3_-N/l [9, 66, 67]. Based on the temperature, pH, and total ammonia nitrogen, the free ammonia in the reactors averaged 445 mg NH_3_-N/l (Supplementary Text). As this level was above reported threshold concentrations for the inhibition of acetoclastic methanogenesis in thermophilic environments, we posit that favorable conditions existed for the conversion of acetate into methane through obligate mutualistic cross-feeding interactions between SAOB and methanogenic archaea.

### Population succession dynamics within acetate-fed enrichment bioreactors

**Fig. 1:**
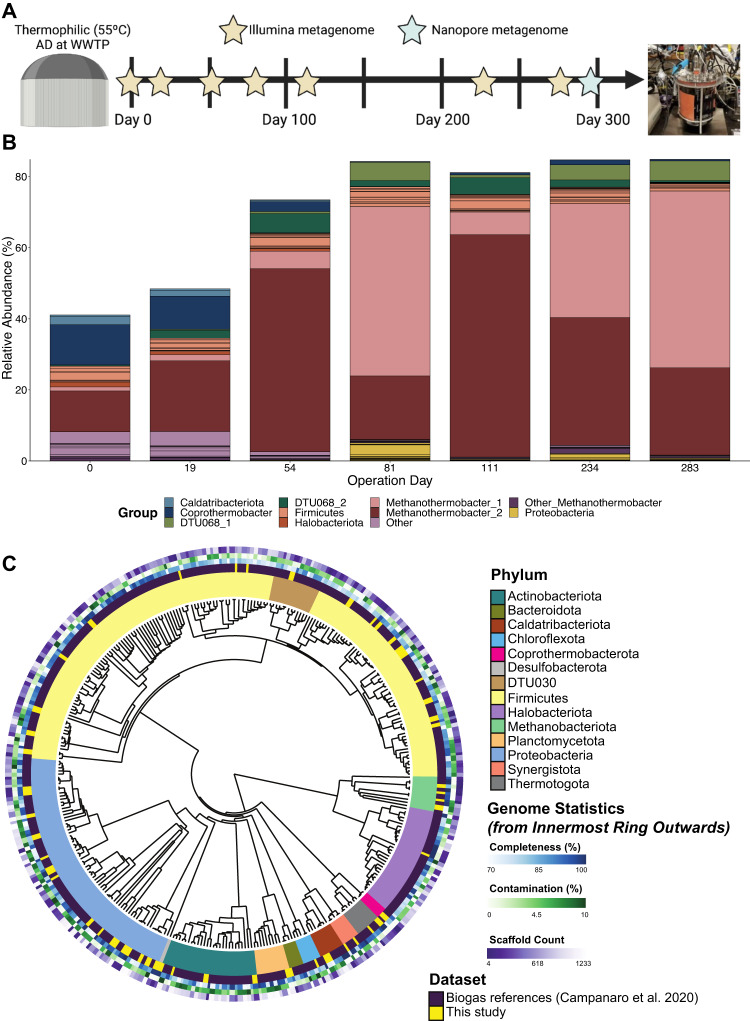
Enrichment of an anaerobic acetate-oxidizing consortium. **A** Conceptual schematic of 300-day chemostat enrichment period of the acetate oxidizing consortium showing the dates that short-read metagenomes were sequenced with Illumina and the long-read metagenome sequenced with Nanopore; **B** The relative read abundance in the short-read metagenomes of the recovered set of de-replicated MAGs within bioreactor R2, which was the bioreactor sampled for the stable isotope probing experiment. The relative read abundance is the number of reads mapped to a given genomic entity divided by the total reads in the sample. Each color in the plot represents a different genomic entity (e.g., a MAG or group of MAGs). **C** Phylogenetic tree of the recovered MAGs and a subset of biogas genome references from Campanaro et al. [69]. Moving from inside to outside in the colored rings: the first (inner) ring shows a phylum level taxonomic classification, the second ring indicates what study the genome originates from, the third ring shows genome completeness, the fourth shows contamination, and the fifth (outer) ring shows the number of scaffolds in the genome. The tree was constructed from ribosomal protein markers with metabolisHMM v2.21 [101] by searching for markers with hmmsearch as part of the HMMER v3.2.1 suite [102], aligning hits for each marker with muscle v3.8.31 [103], and building the phylogenetic tree with fasttree v2.1.11 [104]. The tree was visualized and metadata overlaid on the tree with EMPRESS v1.2.0. [105].

A long-read metagenome assembly of a single bioreactor (R2) community on day 300, as well as individual short-read assemblies of both duplicate bioreactors from 6 operational days over the 300-day period, were used to generate a de-replicated set of 60 medium and high-quality MAGs [68] (Fig. 1 and Supplementary Data 1). The recovered set of MAGs from the enrichment chemostats in this study spanned most of the phyla represented in a previously reported biogas microbiome reference database [69] (Fig. 1C).

Throughout the continuous chemostat operation, the recovered set of 60 de-replicated MAGs was enriched from an initial 40% cumulative read abundance on day 0 to over 85% by day 283 (Fig. 1B and Supplementary Data 1). During the enrichment period, there was a notable washout of MAGs belonging to *Coprothermobacter* and *Caldatribacteriota* (two-tailed Student’s *t* test, days 0–19 (*n* = 4) vs. days 234–283 (*n* = 4); *p* = 4e−5 and 5e−3, respectively) (Fig. 1B). On the other hand, MAGs from the genera DTU068 and *Methanothermobacter_A* significantly increased in abundance throughout the chemostat operation (two-tailed Student’s *t* test, days 0–19 (*n* = 4) vs. days 234–283 (*n* = 4); *p* = 6e−4 and 1e−4, respectively), together increasing from 13% read abundance initially to over 72% after day 81 (Fig. 1B). No archaeal MAGs were recovered belonging to the acetoclastic families (*Methanotrichaceae* and *Methanosarcinaceae*) (Fig. 1B). However, short-read metagenome decomposition using k-mer signatures revealed that the mixotrophic acetoclastic genus, *Methanosarcina*, was initially present at roughly 6% abundance and decreased to below 0.1% by the end of operation (two-tailed Student’s *t* test, days 0–19 (*n* = 4) vs. days 234–283 (*n* = 4); *p* = 8e−3) (Supplementary Fig. S1). Therefore, a highly enriched anaerobic acetate-oxidizing consortium was obtained within 81 days of chemostat operation. The consortium was represented primarily by genomic populations belonging to DTU068 and *Methanothermobacter_A* lineages.

The populations belonging to *Methanothermobacter_A* and DTU068 underwent a dynamic succession over time during the chemostat enrichment. The *Methanothermobacter_2* MAG was initially dominant at 12% read abundance vs. 1% for the *Methanothermobacter_1* MAG, but it was superseded by *Methanothermobacter_1* by the end of the enrichment period (25% vs. 50% read abundance, respectively; Fig. 1B). Along similar lines, the two MAGs belonging to the genus DTU068 also underwent population shifts during the enrichment period. Both DTU068_1 and DTU068_2 MAGs were initially at relatively low read abundances of 0.04% and 0.3%, respectively (Supplementary Data 1). DTU068_2 remained more abundant than DTU068_1 until day 54, reaching 5.5% read abundance vs. 0.6%, respectively. Subsequently, DTU068_1 became the dominant bacterial genome by day 283 reaching 6% read abundance, while DTU068_2 decreased to 0.5% (Fig. 1B). The cause of such population shifts within DTU068 and *Methanothermobacter_A* throughout the enrichment was uncertain, and could potentially be attributed to phage-host dynamics [70] and/or to the establishment of population-specific mutualistic relationships between syntrophic bacteria and methanogens [71].

The *Methanothermobacter_1* and *Methanothermobacter_2* MAGs represented different species with a shared ANI of 83.3% (Supplementary Fig. S2), which is below the 95% ANI cutoff considered for microbial species designation [47]. The *Methanothermobacter_1* MAG shared its highest ANI of 99.5% with *Methanothermobacter_A* sp012840175, while *Methanothermobacter_2* shared its highest ANI of 98.8% with *Methanothermobacter_A* sp003584625 (Supplementary Fig. S2). Notably, these closest relatives of *Methanothermobacter_1* and *Methanothermobacter_2* are so-far uncultured. Within the GTDB taxonomic hierarchy (release 207), the archaeal lineage of *Methanothermobacter* is divided into two genera, *Methanothermobacter_A* and *Methanothermobacter*. Genomes of the representative species *M. tenebrarum* [72] fall within the genus *Methanothermobacter_A*, while *M. marburgensis, M. thermautotrophicus*, and *M. wolfeii* [73, 74] fall within the *Methanothermobacter* genus (Supplementary Fig. S2). These cultured members of *Methanothermobacter* have been characterized to grow optimally at 55 to 65 °C by reducing carbon dioxide to methane using hydrogen, and sometimes formate [73], as electron donors [75]. Several species of *Methanothermobacter* have been isolated from thermophilic municipal sludge anaerobic digesters [73, 74], such as that used as an inoculum source in this study.

Based on genome similarity, DTU068_1 and DTU068_2 MAGs also represented distinct species with 91.6% shared ANI (Supplementary Fig. S3). DTU068_1 shared its highest ANI (99.2%) with DTU068 sp001513545, while DTU068_2 shared its highest ANI (98.6%) with DTU068 sp012840405 (Supplementary Fig. S3). DTU068 represents a so-far uncultured genus within the *Thermacetogeniaceae* family and *Firmicutes* phylum (according to GTDB release 207; *Thermoanaerobacteraceae* family in NCBI taxonomy). MAGs from DTU068 have been hypothesized to participate in SAO based on genome-resolved transcriptomic expression of the Wood-Ljungdahl pathway in a thermophilic (55 °C) manure-fed AD system [76]. To the best of our knowledge, members of DTU068 have not been enriched to the levels observed in this study. In a meta-analysis of 1635 MAGs recovered from 134 anaerobic digestion metagenomes [69], the maximum relative abundance of any DTU068-related MAG was 3.5%, which occurred in the second (methanogenic) phase of a two-phase thermophilic (55 °C) reactor system fed with cheese whey [77]. Based on these observations, the genus DTU068 appears to contain species that thrive in thermophilic anoxic environments and may harbor genes involved in acetate oxidation.

### Time-resolved stable isotope probing metaproteogenomics of enriched community

**Fig. 2:**
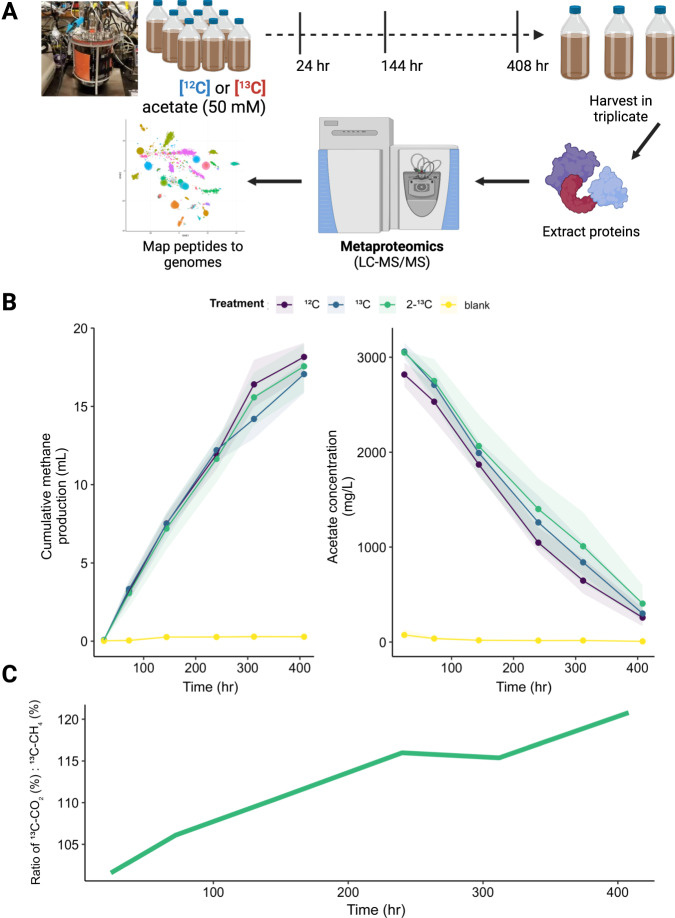
Anoxic stable isotope probing microcosms fed with labeled acetate. **A** Experimental overview of the stable isotope probing (SIP) microcosms and metaproteogenomic analysis; **B** Cumulative methane production and acetate concentrations over time in the SIP microcosms fed with acetate, along with the unfed control (blank). Shaded regions represent the standard error of biological triplicates. **C** Ratio (%) of atom-percent ^13^CO_2_ to that of ^13^CH_4_ measured in the headspace of the SIP microcosms fed with 2-^13^C (methyl-C labeled) acetate, corrected for background ^12^C from dissolved inorganic carbon (see Supplementary Tables S3–S5).

Stable isotope probing with ^13^C-labeled acetate (50 mM; 3000 mg/l) was conducted on the enriched acetate oxidizing members to track their metabolism and identify potential interspecies interactions (Fig. 2A). Over the 408-h incubation, the acetate-fed microcosms produced an average of 17.5 ml CH_4_ (at STP) in excess of the unfed controls (Fig. 2B), corresponding to a theoretical conversion of 88% of the supplied acetate (see Supplementary Text). In concordance with this, the measured acetate concentrations in the microcosms decreased from 3000 mg/l to 280 ± 136 mg/l over 408 h (Fig. 2B and Supplementary Table S2). These results indicate a near-complete conversion of the supplied acetate to CH_4_ in the SIP microcosms, as well as repeatable trends among biological replicates.

The methyl carbon of acetate is converted into CO_2_ during SAO, but reduced to form CH_4_ during acetoclastic methanogenesis [78]. Therefore, we tracked ^13^C:^12^C ratios in the generated CO_2_ and CH_4_ gasses within SIP microcosms fed with 2-^13^C (methyl-C labeled) and 1,2-^13^C (universally labeled) acetate to estimate the relative activity of the SAO pathway vs. acetoclastic methanogenesis [78] (Supplementary Tables S3 and S4). The atom-percent ratio of ^13^CO_2_:^13^CH_4_ in the 2-^13^C acetate-fed microcosms ranged from 1.02 to 1.21 (Fig. 2C and Supplementary Table S4), indicating that most of the methyl carbon was first oxidized to CO_2_ via SAO before being reduced to CH_4_ via hydrogenotrophic methanogenesis. In support of this, we fitted the isotope-partitioning model proposed by Mulat et al. [78] to our experimental data, which suggested that the SAO pathway accounted for 98% ± 5% of the carbon flux from acetate to methane throughout the SIP microcosms (Supplementary Table S5).

**Fig. 3:**
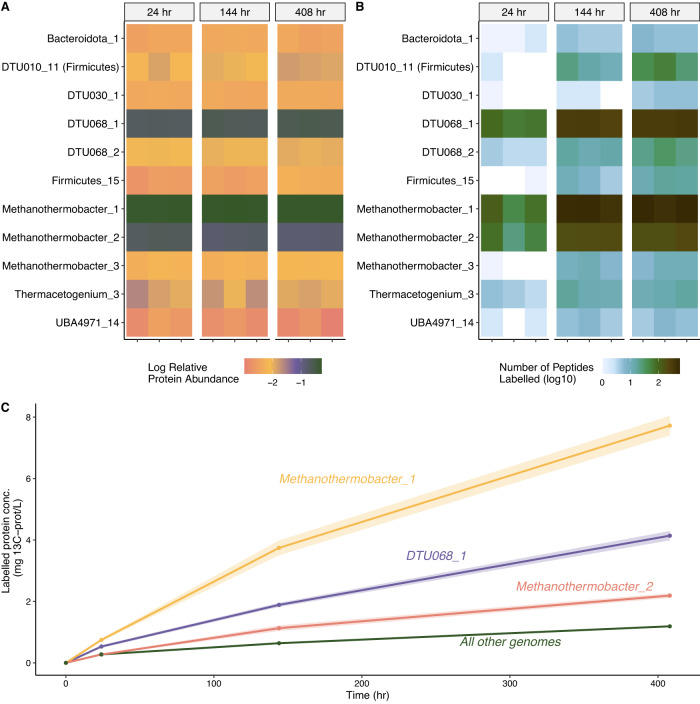
Time-resolved proteomic labeling of SAO consortium within SIP microcosms. Heatmaps of (**A**) relative protein abundance (log_10_-scaled, based on label-free quantification) and (**B**) the number of ^13^C-labeled peptides identified, for the 11 most abundant MAGs at 24, 144, and 408 h of the SIP incubation. Values from biological triplicates are shown for each time point sampled. **C** The ^13^C-labeled protein concentration (mg/L) inferred from the total protein quantification approach, relative isotope abundance (RIA), and labeling ratio (LR) of all proteins from MAGs throughout the SIP incubation. Shaded regions indicate the standard error across biological triplicates, accounting for variance in mean RIA and LR across all proteins in the genome. The MAG names used in this figure are derived from Supplementary Data 1.

At the time of the SIP incubation, metagenomic sequencing indicated that *Methanothermobacter_1* was the most abundant genome (50%) based on fraction of reads mapped, followed by *Methanothermobacter_2* (25%) and DTU068_1 (6%) (Fig. 1B). Correspondingly, 91% ± 4% of the metaproteome was attributed to those three MAGs throughout the SIP incubation, with *Methanothermobacter_1* accounting for 50% ± 1%, followed by DTU068_1 at 23% ± 3% and *Methanothermobacter_2* at 18% ± 3% (Fig. 3A). The extent of isotope incorporation into proteins indicated that the ^13^C atom percent labeling (i.e., relative isotope abundance, RIA) and the abundance of labeled peptides (i.e., labeling ratio, LR) increased across most community members throughout the SIP incubation (Supplementary Fig. S4). Approximately 80% of the identified ^13^C-labeled peptides were mapped to the three members of *Methanothermobacter_1* (39%), *Methanothermobacter_2* (16%), and DTU068_1 (25%) (Fig. 3B). In total, we detected 7879 ^13^C-labeled peptides throughout the 408 h SIP incubation, which is orders of magnitude greater than a previous observation of 61 total ^13^C labeled peptides detected after 196 h of incubation with 100 mM ^13^C-acetate in a mixed anaerobic digestion community [30]. This finding highlights the benefits of long-term community enrichment prior to SIP to gain deeper insights into metabolic activities and carbon flux through SAO populations that are typically rare or in low-abundance in AD systems (Fig. 1B; [20, 24, 65, 79]).

To estimate carbon fluxes into different community members within the SIP incubation, we combined the total protein quantification approach [60] with the ^13^C atom percent labeling (RIA) and labeled-protein abundance (LR) to obtain estimated concentrations of ^13^C-labeled proteins per MAG over time (Fig. 3C). This analysis indicated that *Methanothermobacter_1* produced the most ^13^C-labeled protein (8 mg/L), followed by DTU068_1 (4 mg/L) and *Methanothermobacter_2* (2 mg/L), while all other MAGs accounted for 1 mg/L of ^13^C-labeled protein combined (Fig. 3C). As the ^13^C-protein was directly produced from the added ^13^C-acetate, we estimated an overall community biomass yield for conversion of acetate into methane of 0.01 g-VSS g-acetate^−1^ (0.015 g-COD_biomass_ g-COD_acetate_^−1^), assuming: a protein-to-biomass ratio of 0.5 g-protein g-VSS^−1^ [80], and a VSS-to-COD ratio of 1.42 g-COD_biomass_ g-VSS^−1^. This estimated yield is within the range reported for defined co-cultures of syntrophic propionate-oxidizing bacteria and methanogenic partners of 0.011 to 0.016 g-COD_biomass_ g-COD^−1^ using total protein measurements [81, 82]. Measurements of biomass yields for individual species within syntrophic fatty acid-oxidizing communities are sparse [83], and obtaining absolute biomass estimates for each member separately during growth typically relies on quantitative-PCR [84–86]. Here, we show that quantitatively tracking carbon fluxes into the biomass of individual community members using SIP metaproteomics represents a powerful approach that could help inform ecosystem level models for uncultured microbiomes.

### Metabolic reconstructions and modeling of the SAO community

**Fig. 4:**
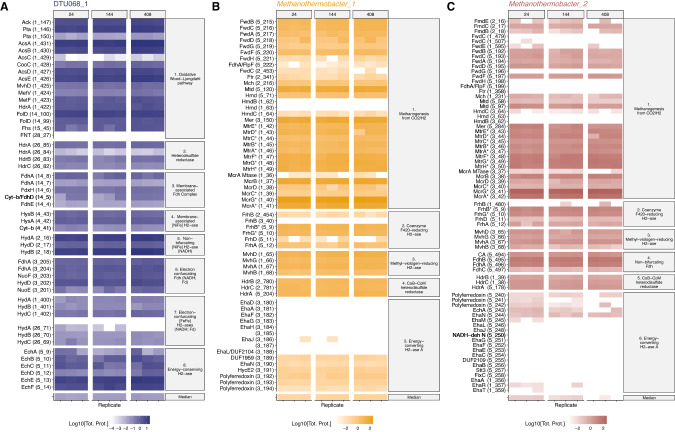
Protein expression of key metabolic pathways in the three-member SAO consortium. Total protein expression (nM; log_10_-scaled) for enzymes of interest throughout the acetate-fed SIP incubations in (**A**) DTU068_1, (**B**) *Methanothermobacter_1*, (**C**) *Methanothermobacter_2*. The vertical facets represent different sampling time points (24, 144, and 408 h), and the horizontal facets represent protein groups based on different metabolic functions and/or protein complexes. The value labeled “Median” at the bottom represents the genome-wide median protein expression. Values are shown for biological triplicates. For each protein unit, the associated gene locus is given in parentheses next to the name. Proteins in *Methanothermobacter_1* and *Methanothermobacter_2* that have an asterisk (*) indicate these associated subunits were identical within the two genomes, and thus the shown protein abundance represents this redundancy. Protein abbreviations: Ack acetate kinase, Acs acetyl-coA synthase/carbon monoxide dehydrogenase (CODH), CooC Acs accessory protein, Cyt cytochrome, DUF domain of unknown function, Ech energy-conserving hydrogenase, Eha energy-converting hydrogenase, Fdh formate dehydrogenase, Fhs formate-THF ligase, Fol methenyl-THF cyclohydrolase, Frh F_420_-reducing hydrogenase, Ftr formyl-MFR:H_4_MPT formyltransferase, Fwd formyl-MFR dehydrogenase, Hdr heterodisulfide reductase, Hmd H_2_-dependent methylene-H_4_MPT dehydrogenase, Hya hydrogenase, Hyd hydrogenase, Mch methenyl-H_4_MPT cyclohydrolase, Mer methylene-H_4_MPT reductase, Mtd F_420_-dependent methylene-H_4_MPT dehydrogenase, MTase methyltransferase, Mtr F_420_-dependent methylene-H_4_MPT dehydrogenase, Mvh F_420_-non-reducing hydrogenase, Mcr methyl-CoM reductase, Nuo NADH:ubiquinone oxidoreductase, Pta phosphotransacetylase.

**Fig. 5:**
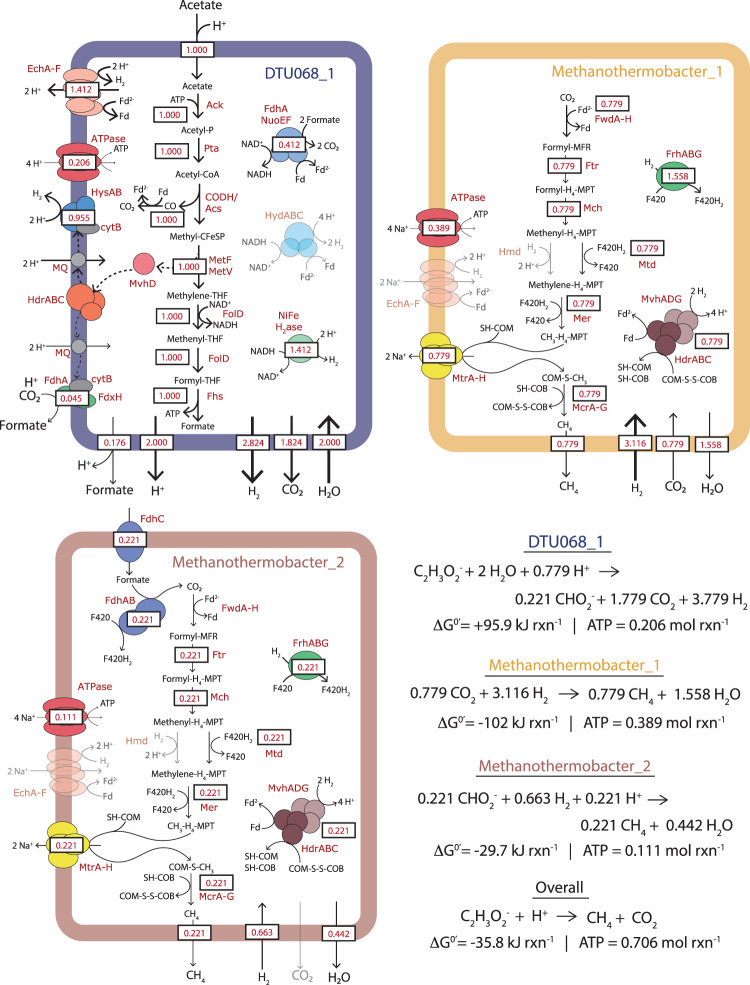
Predicted metabolic fluxes in the three-member SAO consortium. Cell diagrams showing the predicted metabolic pathways for acetate oxidation in DTU068_1 and methane generation from hydrogen/formate in *Methanothermobacter_1* and *Methanothermobacter_2*. Values of predicted flux, obtained from parsimonious flux balance analysis, are shown in red text within boxes. Net catabolic reactions are based on stoichiometry obtained from parsimonious flux balance analysis. Protein abbreviations are defined in the legend of Fig. 4.

Metabolic reconstructions of the three most abundant MAGs (DTU068_1, *Methanothermobacter_1*, and *Methanothermobacter_2*) were created based on predicted functions of expressed proteins (Figs. 4 and 5). In total, DTU068_1 is predicted to oxidize acetate to formate and CO_2_ via the oxidative acetyl-coA pathway (i.e., reverse Wood-Ljungdahl pathway), while producing H_2_ and formate to maintain redox balance. Multiple hydrogen-producing enzymes were found in the DTU068_1 proteome: a NADH-dependent (Group 3b) [NiFe]-hydrogenase ([NiFe]-HydABC), a membrane-bound periplasm-facing (Group 1a) [NiFe]-hydrogenase ([NiFe]-HysAB-Cyt_b_), [FeFe] electron-bifurcating (Group A3) hydrogenases ([FeFe]-HydABC), and a proton-translocating energy-conserving (Group 4e) hydrogenase (EchABCDEF) (Figs. 4 and 5). Formate is predicted to be produced intracellularly through formate-tetrahydrofolate ligase (Fhs), as well as extracellularly via a membrane-associated formate dehydrogenase (Fdh) complex. This membrane-associated Fdh complex and the periplasm-facing [NiFe]-hydrogenase both contain a cytochrome-b subunit (Fig. 4), and are predicted to participate in reverse electron transport from heterodisulfide reductase (HdrABC/MvhD) to drive the endergonic oxidation of methyl-THF to methylene-THF via methylene-THF reductase (MetFV) [87]. An electron-bifurcating FdhA-NuoEF complex was also found that could reversibly oxidize formate to CO_2_ while producing reduced ferredoxin and NADH (Fig. 5). Overall, the predicted pathway for the oxidation of acetate into CO_2_, H_2_, and formate in DTU068_1 was similar to that proposed for *Thermacetogenium phaeum*, except that DTU068_1 is proposed to utilize acetate kinase/phosphotransacetylase (Ack/Pta) to activate acetate to acetyl-coA rather than acetaldehyde oxidoreductase [19], as well as utilize electron-bifurcating FdhA-NuoEF and HydABC complexes for energy conservation (Figs. 4 and 5).

**Fig. 6:**
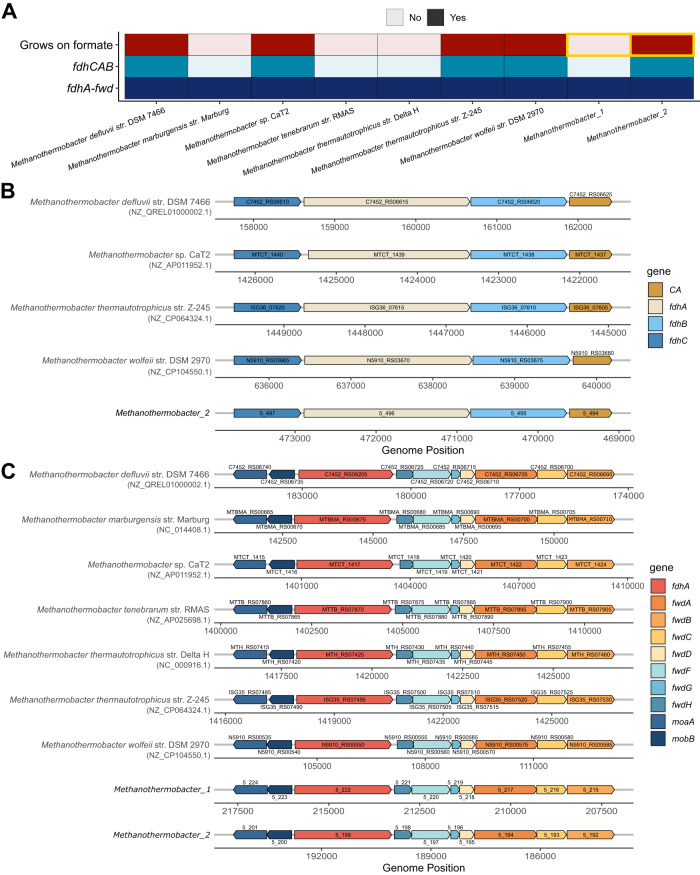
Formate metabolism in *Methanothermobacter* spp. **A** Heatmap showing the ability to grow on formate [73, 75, 106] and the presence/absence of the *fdhCAB* gene cluster and *fdhA-fwd* gene cluster for all available sequenced *Methanothermobacter* genomes, along with the two *Methanothermobacter* MAGs from this study. The yellow borders used for the formate growth values of the two MAGs indicates that these are inferred traits based on the results of this study. **B** Map of the genomic arrangement of the formate dehydrogenase-carbonic anhydrase gene cluster found only within genomes of certain *Methanothermobacter* species, all of which are known to grow on formate, and (**C**) the tungsten formylmethanofuran dehydrogenase gene cluster observed in all available representative *Methanothermobacter* genomes. The gene clusters for the *Methanothermobacter_A* MAGs identified in this study are also shown. The direction of the gene arrows indicate their direction of transcription. NCBI RefSeq accessions of each representative genome is given in parenthesis below its species name. Each gene is labeled with its locus tag, which is sometimes located above/below a gene arrow for visualization purposes. *fwd* tungsten formylmethanofuran dehydrogenase, *fdh* formate dehydrogenase, CA carbonic anhydrase, *moa* GTP 3’,8’-cyclase, *mob* molybdopterin-guanine dinucleotide biosynthesis-domain protein.

Both *Methanothermobacter_1* and *Methanothermobacter_2* contain identical pathways for methane production from CO_2_ (Figs. 4 and 5). A major difference lies in their utilization of formate vs. H_2_ as electron donors. Interspecies electron transport via formate was previously shown to occur during the syntrophic oxidation of acetate [88], propionate [89], and butyrate [90], and has been suggested to permit a greater transfer rate than hydrogen by maintaining feasible thermodynamics across a larger concentration gradient [91]. Formate was detected as one of the most abundant metabolites (besides acetate) in the SIP incubations, ranging in concentration from 3 to 7 μM (Supplementary Table S6). Moreover, the ^13^C-labeling ratio of formate increased over time (Supplementary Table S7), indicating that it was likely a product of acetate oxidation. Within *Methanothermobacter* spp., the ability of *M. thermautotrophicus* Z-245 to grow on formate was attributed to a *fdhAB* gene cluster adjacent to a formate transporter (*fdhC*) and a carbonic anhydrase (*CA*) [92]. The role of this *fdhCAB* gene cluster in growth on formate was recently confirmed by Fink et al. [93] using a shuttle-vector system to amend the canonical non-formate-utilizing *M. thermautotrophicus* ΔH with this operon, which then grew and produced methane from formate. The *fdhCAB* gene cluster was also shown to be essential for growth on formate in the archaeon, *Methanococcus maripaludis* [94]. We queried all sequenced *Methanothermobacter* genomes to-date and found that all species capable of growth on formate as an electron donor possess the *fdhCAB* gene cluster, which was not observed within the genomes of *Methanothermobacter* species incapable of growth on formate (Fig. 6A). *Methanothermobacter_2* was found to contain this *fdhCAB* gene cluster (Fig. 6B), and the FdhABC protein cluster was within the 97 ± 1 percentile of its proteome expression throughout the SIP incubation (Fig. 4). In contrast, *Methanothermobacter_1* did not possess the *fdhCAB* gene cluster (Fig. 6A), and a search of unbinned contigs and unassembled reads confirmed that the only archaeal *fdhC* within the metagenome belonged to *Methanothermobacter_2* (Supplementary Text; Supplementary Tables S8 and S9). While both *Methanothermobacter_1* and *Methanothermobacter_2* MAGs encode for a FdhA unit upstream of the tungsten formylmethanofuran dehydrogenase (*fwd*) gene cluster, we found this *fdhA-fwd* gene arrangement was present in all sequenced *Methanothermobacter* genomes to-date (Fig. 6C), even among members known to not utilize formate as an electron donor like *M. tenebrarum* sp. RMAS [73], *M. thermautotrophicus* ΔH [74], and *M. marburgensis* Marburg [75]. This *fdhA-fwd* gene cluster arrangement was previously reported for the strain *M. thermautotrophicus* ΔH, and the FdhA unit was deemed a “formate dehydrogenase-like protein (FlpF)” due to an N-terminal extension of about 200 amino acids with binding motifs for two [4Fe-4S] clusters [95]. Thus, the function of this FdhA/FlpF enzyme is not clear. Therefore, we predict *Methanothermobacter_2* can oxidize formate via FdhABC to reduce F_420_ for growth, while *Methanothermobacter_1* is predicted to solely grow on H_2_ through its highly expressed methyl-viologen-reducing hydrogenase (MvhABDG) and an F_420_-reducing [NiFe]-hydrogenase (FrhABDG) (Figs. 4 and 5). As DTU068_1 is predicted to produce both H_2_ and formate during the oxidation of acetate (Fig. 5), we posit that this diversity of electron donors could have supported the apparent niche partitioning of methanogenic partners adapted for exclusive or preferential modes of interspecies electron transfer.

To further examine potential syntrophic relationships between DTU068_1, *Methanothermobacter_1*, and *Methanothermobacter_2*, we implemented a metabolic model containing the central carbon and energy metabolism of each guild (Fig. 5). Possible flux distributions were predicted using parsimonious flux balance analysis while constraining the relative ATP yield of each of the three populations to their relative proteome contribution (Fig. 3C). With these constraints, the maximum ATP yield of the entire community was estimated at 0.706 mol ATP mol^−1^ acetate. The metabolic model predicts that DTU068_1 consumes 1 mol acetate and produces 1 mol formate intracellularly. Of the intracellular formate, 0.824 mol is oxidized via an electron-confurcating FdhA-NuoEF complex (Fig. 5). Further, 0.045 mol formate is produced extracellularly using the FdhA-FdxH-CytB complex. The ion motive force (IMF) in DTU068_1 is created by EchABCDEF, and consumed via ATP synthase and to drive reverse electron flow from HdrABC to CO_2_ and H^+^. In total, DTU068_1 is predicted to produce a net of 0.221 mol formate, 1.779 mol CO_2_, and 3.779 mol H_2_ from 1 mol of acetate (Fig. 5).

Overall, the guild-level metabolic model supported the hypothesis that the two methanogens underwent niche partitioning based on their preferred electron donors (e.g., H_2_ or formate). *Methanothermobacter_2* is predicted to consume the 0.221 mol formate produced by DTU068_1, along with 0.663 mol H_2_ (Fig. 5). *Methanothermobacter_1* is predicted to consume 3.116 mol H_2_ and 0.779 mol CO_2_. In both methanogens, H_2_ is consumed via the MvhADG-HdrABC and FrhABG complexes (Fig. 5). Both methanogens also utilize the tetrahydromethanopterin S-methyltransferase complex (MtrABCDEFGH) for IMF generation, which is used for ATP generation with ATP synthase. In total, *Methanothermobacter_1* is predicted to generate 0.779 mol CH_4_ (78% of evolved CH_4_), and *Methanothermobacter_2* is predicted to produce 0.221 mol CH_4_ (22% of evolved CH_4_). The total community ATP production of 0.706 mol ATP and the overall free energy release of −35.8 kJ suggest a net free energy release of −51.1 kJ/mol ATP under standard conditions (Fig. 5), which is likely sufficient to support growth under such energy-limited conditions in anaerobic systems [96, 97].

**Fig. 7:**
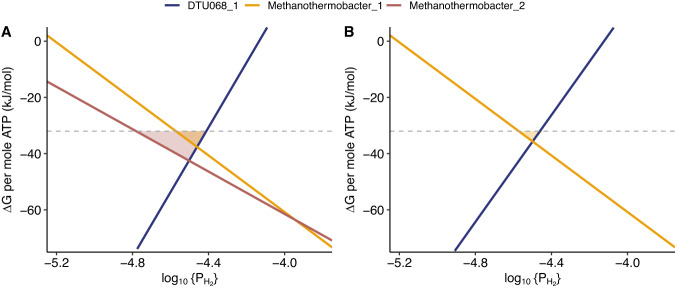
An energetic basis for utilizing diverse electron shuttles during SAO by DTU068_1. Free energy yields (Δ*G*) per mole of ATP produced for each member of the syntrophic acetate oxidizing consortium as a function of hydrogen partial pressure (P_H2_), for the cases where the *Methanothermobacter_2* MAG (**A**) is present; and (**B**) is not present. The dashed gray line represents the ATP phosphorylation potential measured in cells performing acetogenesis from H_2_ and CO_2_ (−32.1 kJ/mol-ATP) [96]. Shaded regions represent the ranges of hydrogen partial pressure that would support ATP synthesis by DTU068_1 and *Methanothermobacter_1* (yellow region) or DTU068_1 and *Methanothermobacter_2* (red region). The free energy values were calculated based on reaction stoichiometry predicted by the parsimonious flux balance analysis model for both cases (Supplementary Data 2), assuming environmentally-relevant concentrations of acetate (50 mM), formate (7.5 μM), methane (0.5atm) and carbon dioxide (0.5atm).

The predicted consumption of intracellular formate by DTU068_1 to generate reduced ferredoxin, along with the consumption of extracellular formate by *Methanothermobacter_2* to drive methanogenesis (Fig. 5), raises the question of whether interspecies electron transfer via formate between these two species represented a mutualistic or competitive interaction. To assess this question, we established a community-scale metabolic model for DTU068_1 grown solely in the presence of *Methanothermobacter_1* (e.g., no *Methanothermobacter_2*) (Supplementary Data 2). As expected, when *Methanothermobacter_2* is not present, DTU068_1 is predicted to consume all of its produced formate via the intracellular electron-bifurcating FdhA-NuoEF complex to generate H_2_, all of which is consumed by *Methanothermobacter_1* to drive methanogenesis (Supplementary Data 2). Interestingly, while the net standard free energy release from 1 mole of acetate of −35.8 kJ is identical, the predicted overall community ATP yield in this scenario is 0.765 moles, which is 8% higher than when *Methanothermobacter_2* is present (Fig. 5). However, an examination of the thermodynamic feasibility of the community metabolism under both scenarios revealed that more favorable energetics for DTU068_1 and *Methanothermobacter_1* are achieved across a wider range of H_2_ partial pressures in the presence of *Methanothermobacter_2* than without (Fig. 7). Thus, interspecies electron transfer via formate from DTU068_1 to *Methanothermobacter_2* does appear to be mutualistic. By shunting electrons to both formate and H_2_ during acetate oxidation, DTU068_1 can establish more favorable energetics for ATP production at the potential sacrifice of net community ATP yield.

While many community-scale models consider the optimization of ATP or biomass yield to be the overarching objective of microbial community metabolism [98, 99], the above finding indicates that anaerobic microbes surviving near thermodynamic limits of life [6] may optimize the energetic favorability of their community metabolism. Previous modeling efforts informed by multi-omics on syntrophic communities of defined isolates have identified electron transfer via different metabolites to be favorable on conditional bases [100]. Our current work using metaproteogenomics-informed SIP builds upon those findings by identifying flexibility in central metabolic processes and electron partitioning that likely governs community composition and fitness through thermodynamic-driven mutualism in so-far uncultured microbes. These insights provide a more nuanced data-driven perspective on community-level modeling of obligate cross-feeding metabolisms driving carbon flux in anoxic ecosystems, such as the case of SAO in AD processes, as well as in the design of synthetic communities for high-value product generation from waste streams.

### Supplementary information

Supplementary Information

Supplementary Data 1

Supplementary Data 2

## Data Availability

All raw metagenomes and de-replicated MAGs are available on NCBI at the Bioproject Accession PRJNA885503 (Supplementary Table S1). Metaproteomic MS data are available on ProteomeXchange as dataset PXD042127. All data files including assemblies, annotated genomes, and metaproteomics results are available on OSF at https://osf.io/kdnms/.

## Code availability

All analysis code, including the metabolic model, is available in the repository: https://github.com/ZielsLab/SAOB_Metaproteogenomics.
