# Supplementary material for: Diverse electron carriers drive syntrophic interactions in an enriched anaerobic acetate-oxidizing consortium
Source: ISME J. 2023 Oct 25;17(12):2326–39. doi: 10.1038/s41396-023-01542-6 (PMC10689502; doi:10.1038/s41396-023-01542-6)
Supplement: Supplementary file 1 — Supplementary Information [file 41396_2023_1542_MOESM1_ESM.docx]

**SUPPLEMENTARY INFORMATION**

**for**

**“Diverse electron carriers drive syntrophic interactions in an enriched anaerobic acetate-oxidizing consortium”**

**SUPPLEMENTARY METHODS**

***Analytical methods for measurements of volatile fatty acids, total and volatile solids, ammonia, pH in enrichment bioreactors***

Total solids (TS) and volatile solids (VS) were determined according to Standard Methods [1]. For Volatile fatty acid (VFA) and total ammonia nitrogen (TAN) analysis, liquid samples were centrifuged at 7,000×g and 10°C for 10 min, and the supernatant solution was filtered through a 0.45 µm PES syringe filter, after which an aliquot of the supernatant was diluted with distilled water to determine TAN concentration by flow injection analyzer (QuickChem FIA+ 8000 Series; Lachat Instruments, USA). A separate filtrate aliquot was further acidified with a drop of 5M HCl to prepare the sample for VFA analysis by gas chromatograph equipped with flame ionization detector (HP 6890 Series, Agilent technologies, Santa Clara, CA, USA).

***Analytical methods for measurements of volatile fatty acids (VFAs) and gas composition in SIP microcosms***

The gas production of all serum bottles was periodically measured with a manometer (Traceable model 3462, VWR, USA). Methane was measured by collecting 6 µL gas samples from the headspace and injecting directly into GC-FID (HP5890 Series ii Gas Chromatograph, Hewlett Packard, USA) with a HP-5 column (Hewlett Packard, USA). Hydrogen was monitored by GC-TCD (Shimadzu GC-2014) with a Hayesep D column, but headspace levels were not detectable via this analysis. VFAs were measured by collecting 0.5 mL liquid samples using a syringe and needle, centrifuging and syringe filtering through a 0.45 µm PES membrane, before injecting on a GC-FID (see above).

Carbon isotope ratios of CH_4_ in stored headspace samples were measured on a Thermo Scientific GasBench II + PreCon trace gas concentration system coupled to a Thermo Scientific Delta V Plus IRMS, located at the Stable Isotope Facility (UC Davis, California). Carbon isotope ratios of headspace CO_2_ were measured on a Thermo Scientific GasBench II coupled to a Thermo Finnigan Delta Plus XL IRMS.

***Determining Free Ammonia Concentration from Total Ammonia, pH, and Temperature***

The concentration of (unionized) free ammonia (NH_3_) is a function of total ammonia nitrogen (TAN), pH, and reactor temperature [2]. The concentration of free ammonia within the enrichment bioreactors was estimated using the formula [2]:


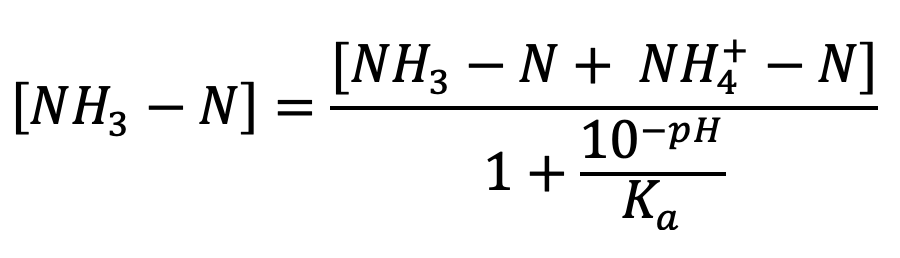


where: [NH_3_-N] is free ammonia, [NH_3_-N + NH_4_^+^-N] is total ammonia nitrogen, and *K_a_* is the acid-base dissociation constant for ammonium. *K_a_* was predicted to be a function of temperature as:


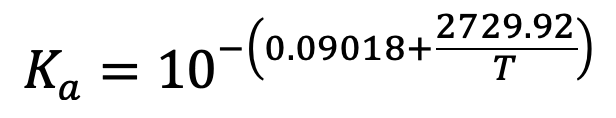


where: T is temperature in Kelvin.

***Preparation of anoxic growth media***

The anoxic growth media used for bioreactor feed and SIP microcosms contained (per 950 mL of media): 0.5 mg resazurin, 0.4 g KH_2_PO_4_, 0.53 g/L Na_2_HPO_4_, 3.82 g/L NH_4_Cl, 0.3 g NaCl, 0.1 g/L MgCl_2_ 6H_2_O, 50 mM HCl, 1mM H_3_BO_3_, 0.5 mM MnCl_2_, 7.5 mM FeCl_2_ , 0.5 mM CoCl_2_, 0.1 mM NiCl_2_, 0.5 mM ZnCl_2_, 10 mM NaOH, 0.1 mM Na_2_SeO_3_, 0.1 mM Na_2_WO_4_, and 0.1 mM Na_2_MoO, dissolved in distilled water. The above solution was boiled to remove O_2_, cooled under a constant flow of N_2_, dispensed into serum bottles sealed with butyl rubber septa, and autoclaved. After autoclaving, the following filter-sterilized stocks were added to the solution (per 950 mL boiled volume): 10 mL CaCl_2_ (11 g/liter); 1 ml vitamin solution. The vitamin stock solution contained (per liter final medium): 20 mg biotin, 200 mg nicotinamide, 100 mg p‐aminobenzoic acid, 200 mg thiamin, 100 mg panthothenic acid, 500 mg pyridoxamine, 100 mg cyanocobalamin (vitamin B12 ), and 100 mg riboflavin. Sodium acetate was added to the reach desired concentration (e.g. 75 mM for bioreactor feed, or 500 mM for SIP microcosm stocks). Finally, to reduce the media further, 50 ml NaHCO_3_ filter-sterilized solution (80 g/liter), 1 ml Na_2_H_2_O filter-sterilized solution (240 g/liter), 0.5 g cystein-HCl were added.

***Estimating substrate conversion efficiency in SIP batch tests***

The substrate conversion efficiency in the SIP batch tests was estimated based on a mass balance on chemical oxygen demand (COD). 1 mol of CH_4_ possesses a COD equivalent of 64 g (e.g. 2 moles (64 g) of O_2_ are consumed per 1 mol CH_4_ combusted to CO_2_). At standard temperature and pressure, 1 mole of CH_4_ occupies 22.4 l, meaning the specific volume of CH_4_ is 0.35 l/g-COD. Ignoring COD assimilation into biomass, the COD mass balance on substrate conversion is:


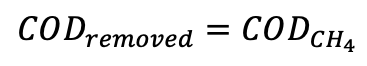


where: *COD_removed_* is the amount of acetate COD consumed, and *COD_CH4_* is the amount of COD as CH_4_ produced. As acetate has a COD content of 0.95 g-COD/g-acetate, and 0.06 g acetate was added to each SIP bottle (3 g/l into 20 ml), the theoretical maximum amount of CH_4_ that could be produced in each microcosm bottle was 19.95 ml.

***Protein Extraction***

Samples (200 uL) were transferred to a 1.5-mL Eppendorf Biopur tube with an equal amount of 0.1 mm Zirconia/Silica beads and 2X sample volume of 100 mM ammonium bicarbonate buffer, pH 8.0. Samples were lysed in a Bullet Blender (NextAdvance, Troy, NY) for 3 minutes at 4C, speed of 8. The lysate was collected by poking a hole in the base of the 1.5-mL tube with a 26-Ga needle and nesting that tube into a 15-mL centrifuge tube followed by centrifuging at 4C, 4500 x g for 5 minutes. A BCA protein assay (ThermoFisher Scientific, Waltham, MA, USA) was performed to determine approximate protein concentration of each sample and the volume of the samples were normalized. Urea and dithiothreitol (ThermoFisher Scientific, Waltham, MA, USA) was added to a 8M and 5 mM concentration (respectively) and the samples were incubated on a Thermomixer (Eppendorf) for 30 min at 60C with shaking at 850 rpm. Samples were diluted eight-fold with 1.14 mM CaCl2 in 50 mM ammonium bicarbonate and trypsin (Promega; Madison, WI, USA) was added in a 1:50 (trypsin:protein, w:w) ratio. The samples were incubated for 3 hours at 37C with shaking at 850 rpm, then frozen and stored at -70C. Solid Phase Extraction (SPE) was performed on the samples using 1 mL/50 mg C18 columns from Phenomemex (Torrance, CA). Columns were conditioned with 3 mL of methanol followed by 2 mL of 0.1% trifluoroacetic acid (TFA) in water. The samples were applied to individual columns followed by 4 mL of 95:4.9:0.1 H2O:MeCN:TFA. Samples were eluted into 1.5-mL microcentrifuge tubes with 1 mL of 80:19.9:0.1 MeCN:H2O:TFA and concentrated in a vacuum concentrator to 75 uL. Another BCA protein assay was performed to obtain peptide concentration and a portion of each sample was diluted to 0.1 ug/uL for LC-MS analysis.

***LC-MS/MS for metaproteomics analysis***

MS analysis was performed using a Q‐Exactive HF-X mass spectrometer (Thermo Scientific) by acquiring datasets for liquid chromatographic separation of a 0.1 ug/uL of peptide solution. The ion transfer tube temperature and nano electrospray voltage were 300°C and 2.2 kV, respectively. Each dataset was collected for 120 min following a 10 min delay after completion of sample trapping and start of gradient. FT‐MS spectra were acquired from 300 to 1800 m/z at a resolution of 60 k (AGC target 3e6) and the top 12 FT‐HCD‐MS/MS spectra were acquired in data‐dependent mode with an isolation window of 0.7 m/z at a resolution of 45 k (AGC target 1e5). A normalized collision energy of 30 was used for HCD fragmentation, with a 45 s exclusion time, analyzing only charge states 2 to 6. Liquid chromatography separations were done using a Thermo Dionex Ultimate, configured with 2 pumps for sample trapping and reverse-flow elution of the sample onto the analytical column respectively.

***Bioinformatics for metaproteomics data analysis***

The MS/MS spectra were searched using the MS-GF+ tool [3] against a protein database consisting of all ORFs from the set of de-replicated polished MAGs, concatenated with reversed (decoy) sequences of all protein entries for the estimation of false discovery rates (FDRs). Mass tolerances were set to 10 ppm for precursor ions and 0.5 Da for fragment ions, respectively, and unlimited missed cleavages of trypsin were allowed in the database searching. Carbamidomethylation of cysteine residues was used as a fixed modification, while the oxidation of methionine was a variable modification. Peptide spectra matches (PSMs) were indexed and extracted with the ‘PeptideIndexer’ and ‘PSMFeatureExtractor’ tools in OpenMS, and were filtered at a 5% FDR threshold following error probability estimation with Percolator [4]. Filtered PSMs were then used to identify features in the MS1 data using the ‘FeatureFinderIdentification’ algorithm [5].

For label-free quantification (LFQ) of proteins, PSMs from unlabeled (^12^C) samples were used for protein inference with Fido [6], followed by protein FDR filtering at 5%. The retention time of annotated features in the unlabeled proteomes were aligned using ‘MapAlignerIdentification’ in OpenMS, and were grouped using ‘FeatureLinkerUnlabeledQT’. Ambiguous annotations of shared features were resolved with ‘IDConflictResolver’. Quantification of proteins was performed with ‘ProteinQuantifier’ based on the summed intensities of all PSMs within an inferred protein group, and only proteins with unique PSMs were used in subsequent aggregation and quantitative analysis.

To identify labeled peptides in the MS data, we used the MetaProSIP algorithm [7] implemented through OpenMS. As isotope incorporation can hinder peptide identification due to mass shifts [7], we leveraged the MS data from the triplicate unlabeled (^12^C) biological controls to aid in the identification of unlabeled peptides, which were then queried for isotope incorporation within the labeled (^13^C) samples. The retention times of filtered peptides in a single labeled sample were aligned to those in triplicate unlabeled samples from the same time point using ‘MapAlignerIdentification’ and ‘IDMerger’ in OpenMS. The merged peptide identifications were then used, along with the MS/MS spectra of the single labeled sample, as input to ‘FeatureFinderIdentification’ to detect features in the MS1 data. The annotated features were then used to quantify isotope incorporation into peptides using the MetaProSIP algorithm using default settings.

***NMR for metabolomics analysis***

Filtered (0.2 µm Titan PTFE syringe filters (Thermo Scientific, USA)) chemostat supernatants for NMR metabolomics analysis were aliquoted into 0.180 mL and 3.0 mL portions with the former measured directly (as described below) after adding 0.020 mL Chenomx standard solution (containing 5.0 mM DSS-d6, 0.01 % (w/v) sodium azide, in 100% D_2_O, Chenomx Inc., Edmonton, AB) and transferring to Bruker 3.0 mm O.D. glass NMR tubes. The 3.0 mL portions were flash frozen in liquid nitrogen, lyophilized to dryness, and reconstituted in 0.300 mL of D2O (comprised of 10% Chenomx standard solution) to give a 10-fold concentration increase thus facilitating the measurement of lower abundance non-volatile metabolites as well as aiding in ^13^C isotope analysis for a subset of the same (formate, glycolate, propionate, and succinate) in the 1,2-^13^C2-acetate supplied experiments as described below. Two-hundred microliters of each concentrated supernatant were transferred to separate 3.0 mm NMR tubes for measurement. All NMR spectra were measured at a field strength of 17.6 T (750.2 MHz ^1^H) on a Bruker Avance III spectrometer equipped with a Bruker 5mm HCN TCI CryoProbe with Z-gradient. Sample temperature was regulated at 298.0 K. The 1D ^1^H NOESY experiment with low power, continuous wave pre-saturation of the water signal (Bruker pulse program ‘noesypr1d’) was acquired for all samples with the following parameters, spectral width of 12.0 ppm (9.0 kHz), a relaxation delay of 1.5 s, a mixing time of 100 ms, an acquisition time of 4.0 s (72070 total points), and a total of 2048 transients were coadded for each spectrum. Post-acquisition processing included zero-filling to 262144 real points and multiplication of the FID by a decaying exponential (0.5 Hz line-broadening) using Bruker’s Topspin 3.6.3. Spectra were imported into Chenomx NMR Suite version 9.0 Professional (Chenomx Inc., Edmonton, AB) for final pre-processing, including baseline correction (Chenomx spline algorithm), and metabolomic profiling (see Table S6 for results). Additionally, the DISPEL (Destruction of Interfering Satellites by Perfect-Echo Low-pass filtration) experiment was employed for the ten-fold concentrated labeled acetate supernatant samples to identify ^13^C satellites and estimate site-specific incorporation in glycolate, propionate, and succinate specifically as their signals resided in heavily overlapped regions of the spectrum. In brief, the experiment measures both a standard and a one-bond ^1^H-^13^C satellite suppressed spectrum. Subtraction of the ^13^C-satellite suppressed spectrum from the standard spectrum yields a difference spectrum containing only one-bond ^1^H-^13^C satellites which vastly simplifies both identification and quantitation of their contributions to the overall metabolite signal. The four-stage DISPEL pulse program with continuous wave low power pre-saturation of the water signal [9], available from the Manchester NMR methodology group website (https://www.nmr.chemistry.manchester.ac.uk/?q=node/437), was employed with the added feature of a 4-stage interleaved acquisition of the reference and ^13^C satellite suppressed transients courtesy of, and pending publication by, Peat et al. [10] Experimental parameters employed included a spectral width of 20.2 ppm (16 kHz), acquisition time of 4.1 s (131072 total points), a relaxation delay of 3.0 s (during which low-power continuous wave pre-saturation of the water signal was applied), and a total of 1024 transients were acquired per each spectrum (reference and one-bond ^13^C satellite suppressed) following 4 steady state transients for a total experiment time of 4.1 h. Post-acquisition processing included zero-filling to 262144 real points and multiplication by a decaying exponential function (EM, line broadening of 0.3 Hz) prior to Fourier transform. Additionally, phase correction, and subtraction of the reference and suppressed ^13^C satellite spectra were performed on the instrument using Topspin 3.6.3. All further processing, including baseline correction and spectral deconvolution, were performed using MNova 14.0.1 (Mestrelab Research S. L). Isotopologue fractions determined via integrations of ^13^C satellite and parent ^12^C peak areas are compiled in Table S7 and corrections to the solutions concentrations have been applied where applicable in Table S6.

***Annotation of bifurcating formate dehydrogenases and hydrogenases***

To determine whether formate dehydrogenase (Fdh) and/or hydrogenase complexes were electron bifurcating or not, we utilized both automated database searches along with manual inspection. For enzyme clusters that were initially annotated as hydrogenases, we queried the amino acid sequences with the HydDB [8], which classifies units as electron bifurcating or not. As HydDB does not classify Fdh clusters, we manually queried the beta subunits of those for the amino acid motifs identified by Losey et al. [9] for classification of electron bifurcating units. Specifically, electron-bifurcating beta subunits were characterized by the criteria [9]: (1) the presence of the motif, ‘[A/E]FM’ instead of ‘[T/S][F/Y][K/S/A]’, approximately 5 positions down from the conserved ‘N[A/G/V]DE’ motif in the NADH-binding domain; (2) the substitution of ‘F’ for ‘Y’ in-between the sequence motifs ‘GAGA’ and ‘CGE’ in the FMN-binding domain; (3) the presence of the motif ‘GGPSG’ in the SLBB-binding domain. Beta-units of electron-bifurcating hydrogenases predicted by HydDB were also manually inspected for the above classification criteria.

***Querying metagenomes for possible formate transporter genes of Methanothermobacter_1***

We investigated the possibility that *Methanothermobacter_1* could have possessed a formate transporter gene that was initially undetected because it was unbinned or remained unassembled in the metagenome. For this inquiry, we searched the polished long-read metagenome assembly from day 300, as this assembly had a 100% metagenome read recruitment rate, indicating that there essentially was no information loss from unassembled reads. Initially, homologous genes encoding for the formate transporter utilized by *Methanothermobacter*, *fdhC*, within the long-read assembly were searched using a hidden Markov model (HMM) against a reference profile HMM database made from *fdhC* genes from the genomes shown in Table S10. The reference *fdhC* amino acid sequences were aligned using MUSCLE (v5.1) and a profile HMM was created using HMMER (v3.1b2). Open reading frames in the metagenome were then searched using this profile HMM via the program ‘hmmsearch’ with a threshold E-value of 10^-3^ (Supplementary Table S8). Following detection via HMM, we further verified the potential *fdhC* genes and their taxonomic placement using BLASTp against the NCBI nr database (May, 2023) (Supplemental Table S9). The results indicated that the only archaeal *fdhC* gene in the metagenome was binned with *Methanothermobacter_2*, and thus it is unlikely that *Methanothermobacter_1* lacks this gene due to binning or assembly errors.

We also verified that the genome annotation of *Methanothermobacter_1* from MetaPathways did not predict any alternative formate transporters. We additionally performed a BLASTp search of all ORFs within the polished long-read metagenome assembly against *focA* (NCBI Accession: CAD6012001.1) and *focB* (NCBI Accession: CAD6000752.1) genes from *Escherichia coli*, and did not observe any archaeal ORFs that had matches to those genes (over 50% amino acid identity and an E-score less than 1e-3). These findings suggest that it is unlikely that alternative formate transporters that belonged to *Methanothermobacter_1* remained unbinned in its genome. In other words, this supports the notion that *Methanothermobacter_1* lacked a mechanism for formate transport.

**Table S1:** Summary of metagenomic sequencing metadata, throughput, and NCBI accessions

| **Reactor** | **Operating Day** | **Sequence Type** | **Sequencing Throughput (Gbp)** | **NCBI BioSample Accession** |
| --- | --- | --- | --- | --- |
| R1 | 0 | Illumina | 10.5 | SAMN33819454 |
| R1 | 19 | Illumina | 12.4 | SAMN33819449 |
| R1 | 54 | Illumina | 14.8 | SAMN33819451 |
| R1 | 81 | Illumina | 21.9 | SAMN33819450 |
| R1 | 111 | Illumina | 13.3 | SAMN33819453 |
| R1 | 234 | Illumina | 25.3 | SAMN33819452 |
| R1 | 283 | Illumina | 15.2 | SAMN33819455 |
| R2 | 0 | Illumina | 20.3 | SAMN33819461 |
| R2 | 19 | Illumina | 15.6 | SAMN33819456 |
| R2 | 54 | Illumina | 15.8 | SAMN33819458 |
| R2 | 81 | Illumina | 21.6 | SAMN33819457 |
| R2 | 111 | Illumina | 14.7 | SAMN33819460 |
| R2 | 234 | Illumina | 24.4 | SAMN33819459 |
| R2 | 283 | Illumina | 24.6 | SAMN33819462 |
| R2 | 300 | Nanopore (R10.4) | 10.8 | SAMN35100041 |

**Table S2:** Acetate concentrations in the SIP microcosms over time, measured by GC-FID. Mean and standard deviations refer to measurements from triplicate biological replicates.

| **Time (hr)** | **Treatment** | **Mean Acetate (mM)** | **Std. dev (mM)** |
| --- | --- | --- | --- |
| 24 | ^12^C | 48 | 3.6 |
| 24 | ^13^C | 52 | 1.5 |
| 72 | ^12^C | 43 | 3.2 |
| 72 | ^13^C | 46 | 2.2 |
| 144 | ^12^C | 32 | 3.2 |
| 144 | ^13^C | 34 | 3.1 |
| 240 | ^12^C | 18 | 2.4 |
| 240 | ^13^C | 21 | 4.4 |
| 312 | ^12^C | 11 | 3.5 |
| 312 | ^13^C | 14 | 3.2 |
| 408 | ^12^C | 4.4 | 2.3 |
| 408 | ^13^C | 5.1 | 2.3 |

**Table S3:** Atom percent distribution of ^13^C and ^12^C labeled CO_2_ and CH_4_ within the headspace of the SIP microcosms amended with universally labeled [^13^C]-acetate (i.e. [1,2-^13^C]-CH_3_COO^-^), measured via IRMS. These values were used to correct for [^12^C]-CO_2_ and [^12^C]-CH_4_ production from background inorganic carbon in the inoculum within the set of microcosms incubated with methyl-labeled [^13^C]-acetate (see Supplemental Table S4).

| **Time (hr)** | **[1,2-^13^C]-CH_3_COO^-^ incubation (for background TIC correction)** | | | | |
| --- | --- | --- | --- | --- | --- |
|  | Biological Replicate | atom-% ^13^C-CO_2_ | atom-% ^12^C-CO_2_ | atom-% ^13^C-CH_4_ | atom-% ^12^C-CH_4_ |
| 24 | 1 | 2.200 | 97.800 | 4.937 | 95.063 |
|  | 2 | 3.176 | 96.824 | 5.525 | 94.475 |
|  | 3 | 3.863 | 96.137 | 6.415 | 93.585 |
| 72 | 1 | 4.776 | 95.224 | 14.000 | 86.000 |
|  | 2 | 6.382 | 93.618 | 15.692 | 84.308 |
|  | 3 | 8.252 | 91.748 | 17.284 | 82.716 |
| 144 | 1 | 7.533 | 92.467 | 23.780 | 76.220 |
|  | 2 | 10.655 | 89.345 | 25.398 | 74.602 |
|  | 3 | 12.686 | 87.314 | 26.955 | 73.045 |
| 240 | 1 | 8.400 | 91.600 | 32.642 | 67.358 |
|  | 2 | 12.585 | 87.415 | 34.434 | 65.566 |
|  | 3 | 15.939 | 84.061 | 36.006 | 63.994 |
| 312 | 1 | 9.738 | 90.262 | 37.956 | 62.044 |
|  | 2 | 14.121 | 85.879 | 39.154 | 60.846 |
|  | 3 | 16.177 | 83.823 | 38.767 | 61.233 |
| 408 | 1 | 10.690 | 89.310 | 41.966 | 58.034 |
|  | 2 | 15.188 | 84.812 | 43.045 | 56.955 |
|  | 3 | 18.635 | 81.365 | 44.615 | 55.385 |

**Table S4:** Atom percent distribution of ^13^C and ^12^C labeled CO_2_ and CH_4_ within the headspace of the SIP microcosms amended with methyl-labeled [^13^C]acetate (i.e. [2-^13^C]-CH_3_COO^-^), measured via IRMS. At each time point, the mean ^12^C-CO_2_ and ^12^C-CH_4_ atom-percentages from the microcosms incubated with universal ^13^C acetate (Supplemental Table 3) were subtracted from those in the methyl-labeled ^13^C acetate samples to obtain values corrected for ^12^C-CO_2_ and ^12^C-CH_4_ production from background total inorganic carbon in the inoculum (i.e. BG TIC Corrected), per [10].

| **Time (hr)** | **[2-^13^C]-CH_3_COO^-^ incubation** | | | | | | | | | |
| --- | --- | --- | --- | --- | --- | --- | --- | --- | --- | --- |
|  | Biological Replicate | atom-% ^13^C-CO_2_ | atom-% ^12^C-CO_2_ | atom-% ^12^C-CO_2_ (BG TIC corrected) | atom-% ^13^C-CO_2_ (BG TIC corrected) | atom-% ^13^C-CH_4_ | atom-% ^12^C CH_4_ | atom-% ^12^C-CH_4_ (BG TIC corrected) | atom-% ^13^C-CH_4_ (BG TIC corrected) | ^13^CO_2_/^13^CH_4_ (BG TIC corrected) |
| 24 | 1 | 1.698 | 98.302 | 1.30 | 98.70 | 2.990 | 97.010 | 2.82 | 97.18 | 102% |
|  | 2 | 1.892 | 98.108 |  |  | 2.958 | 97.042 |  |  |  |
|  | 3 | 1.757 | 98.243 |  |  | 2.458 | 97.542 |  |  |  |
| 72 | 1 | 2.580 | 97.420 | 3.29 | 96.71 | 7.236 | 92.764 | 8.85 | 91.15 | 106% |
|  | 2 | 3.378 | 96.622 |  |  | 7.230 | 92.770 |  |  |  |
|  | 3 | 3.586 | 96.414 |  |  | 5.956 | 94.044 |  |  |  |
| 144 | 1 | 3.099 | 96.901 | 5.53 | 94.47 | 11.281 | 88.719 | 14.36 | 85.64 | 110% |
|  | 2 | 5.083 | 94.917 |  |  | 11.642 | 88.358 |  |  |  |
|  | 3 | 6.096 | 93.904 |  |  | 10.145 | 89.855 |  |  |  |
| 240 | 1 | 4.404 | 95.596 | 5.91 | 94.09 | 15.545 | 84.455 | 18.88 | 81.12 | 116% |
|  | 2 | 6.711 | 93.289 |  |  | 16.159 | 83.841 |  |  |  |
|  | 3 | 8.085 | 91.915 |  |  | 14.752 | 85.248 |  |  |  |
| 312 | 1 | 4.713 | 95.287 | 7.54 | 92.46 |  |  | 19.86 | 80.14 | 115% |
|  | 2 | 6.899 | 93.101 |  |  | 18.764 | 81.236 |  |  |  |
|  | 3 |  |  |  |  |  |  |  |  |  |
| 408 | 1 | 5.900 | 94.100 | 6.92 | 93.08 | 20.423 | 79.577 | 22.94 | 77.06 | 121% |
|  | 2 | 8.184 | 91.816 |  |  | 20.482 | 79.518 |  |  |  |
|  | 3 | 9.681 | 90.319 |  |  | 19.916 | 80.084 |  |  |  |

**Table S5:** Modeled values of intermediate metabolites based on the consumption of 100 mole of methyl labelled acetate (e.g. [2-^13^C]-CH_3_COO^-^), based on the theoretical model proposed by Mulat et al. [10]. This model accounts for stoichiometry of product consumption and production in acetoclastic methanogenesis (AM), syntrophic acetate oxidation (SAO), and hydrogenotrophic methanogenesis (HM). Values in columns with those designations (AM, SAO, HM, sum, unconsumed) are in units of mol. Kinetic inhibition effects (KIE) of ^13^C-labeled substrates are also accounted for in the HM pathway. The values of the model-predicted ^13^CO_2_:^13^CH_4_ ratios were fit to the observed ratios (corrected for background inorganic carbon, see Supplemental Table S4) by changing the percent SAO activity relative to AM, using Excel Solver.

| **Time (hr)** | **Atom % ^13^CO_2_/ ^13^CH_4_ (Actual)** | **Model values for 100 mol of [2-^13^C]-CH_3_COO^-^ transformation (used to fit % SAO activity to observed ^13^CO_2_/^13^CH_4_ values)** | | | | | | | | | | | | | | | | |
| --- | --- | --- | --- | --- | --- | --- | --- | --- | --- | --- | --- | --- | --- | --- | --- | --- | --- | --- |
|  |  | % SAO Activity | % AM Activity | ^13^CH_4_ (AM) | ^12^CO_2_ (AM) | ^12^CO_2_ (SAO) | ^13^CO_2_ (SAO) | H_2_ (SAO) | ^12^CO_2_ (sum) | ^12^CH_4_ (HM) | ^13^CH_4_ (HM) | ^13^CH_4_ (HM, KIE) | ^12^CH_4_ (HM, KIE) | ^12^CO_2_ (uncon-sumed) | ^12^CO_2_ (uncon-sumed) | %^13^CO_2_ | %^13^CH_4_ | Model atom % ^13^CO_2_/ ^13^CH_4_ |
| 24 | 1.02 | 90% | 10% | 10.2 | 10.2 | 89.8 | 89.8 | 359.2 | 100.0 | 47.3 | 42.5 | 39.4 | 50.4 | 49.6 | 50.4 | 0.5 | 0.5 | 1.02 |
| 72 | 1.06 | 94% | 6% | 6.4 | 6.4 | 93.6 | 93.6 | 374.3 | 100.0 | 48.3 | 45.2 | 42.1 | 51.5 | 48.5 | 51.5 | 0.5 | 0.5 | 1.06 |
| 144 | 1.10 | 97% | 3% | 3.0 | 3.0 | 97.0 | 97.0 | 388.2 | 100.0 | 49.2 | 47.8 | 44.6 | 52.4 | 47.6 | 52.4 | 0.5 | 0.5 | 1.10 |
| 240 | 1.16 | 102% | -2% | -1.7 | -1.7 | 101.7 | 101.7 | 406.8 | 100.0 | 50.4 | 51.3 | 48.0 | 53.7 | 46.3 | 53.7 | 0.5 | 0.5 | 1.16 |
| 312 | 1.15 | 101% | -1% | -1.2 | -1.2 | 101.2 | 101.2 | 404.8 | 100.0 | 50.3 | 50.9 | 47.6 | 53.6 | 46.4 | 53.6 | 0.5 | 0.5 | 1.15 |
| 408 | 1.21 | 104% | -4% | -4.0 | -4.0 | 104.0 | 104.0 | 416.0 | 100.0 | 51.0 | 53.0 | 49.7 | 54.3 | 45.7 | 54.3 | 0.5 | 0.5 | 1.19 |

**Table S6:** Metabolites in filtered and lyophilized liquid samples from the SIP microcosms over time, measured by NMR. Concentrations are in units of μM. Mean and standard deviations refer to measurements from triplicate biological replicates. For each time point, ‘^12^C’ and ‘^13^C’ columns refer to triplicate incubations with unlabeled or universally ^13^C-labeled acetate, respectively.

| **Time** | **24 hr** | | **144 hr** | | **408 hr** | |
| --- | --- | --- | --- | --- | --- | --- |
| **Treatment** | **^12^C** | **^13^C** | **^12^C** | **^13^C** | **^12^C** | **^13^C** |
| **Mean Acetate** | 37489 | 38645 | 14601 | 17036 | 2335 | 2775 |
| **Stdev Acetate** | 4559 | 13912 | 3423 | 6398 | 522 | 1260 |
| **Mean Ethanol** | 12 | 7.3 | 3.7 | 5.8 | 2.5 | 4.1 |
| **Stdev Ethanol** | 9.5 | 6.5 | 2.2 | 3.9 | 1.7 | 2.3 |
| **Mean Formate** | 7.3 | 3.8 | 3.3 | 5.0 | 5.5 | 3.9 |
| **Stdev Formate** | 0.2 | 0.9 | 1.0 | 1.6 | 0.7 | 0.6 |
| **Mean glycolate** | 2.9 | 4.6 | 1.1 | 2.8 | 0.3 | 0.6 |
| **Stdev Glycolate** | 0.2 | 1.7 | 0.1 | 0.6 | 0.1 | 0.5 |
| **Mean Isovalerate** | 1.2 | 1.3 | 1.3 | 1.6 | 2.2 | 2.0 |
| **Stdev Isovalerate** | 0.3 | 0.5 | 0.4 | 0.6 | 0.5 | 0.5 |
| **Mean Methanol** | 4.8 | 4.5 | 4.1 | 4.2 | 3.9 | 5.0 |
| **Stdev Methanol** | 0.8 | 0.8 | 0.1 | 0.3 | 0.1 | 1.5 |
| **Mean Propionate** | 49 | 47 | 34 | 44 | 45 | 53 |
| **Stdev Propionate** | 13 | 18 | 15 | 24 | 15 | 9.5 |
| **Mean Succinate** | 0.8 | 0.9 | 3.0 | 4.3 | 1.2 | 5.8 |
| **Stdev Succinate** | 0.7 | 0.1 | 1.9 | 2.2 | 0.6 | 0.5 |

**Table S7:** Labeled metabolites based on analysis of NMR spectra lyophilized filtered samples from the SIP microcosm experiments (triplicate biological replicates at each time point). The fraction of different isotopologues are shown for detected labeled metabolites.

| **Isotopologue** | **24 hrs** | | | **144 hrs** | | | **408 hrs** | | |
| --- | --- | --- | --- | --- | --- | --- | --- | --- | --- |
|  | Rep.1 | Rep.2 | Rep.3 | Rep.1 | Rep.2 | Rep.3 | Rep.1 | Rep.2 | Rep.3 |
| **Acetate** | | | | | | | | | |
| ^12^C1, ^12^C2 | 0.042 | 0.038 | 0.051 | 0.103 | 0.092 | 0.080 | 0.130 | 0.127 | 0.168 |
| ^13^C1, ^12^C2 | 0.009 | 0.008 | 0.009 | 0.030 | 0.036 | 0.020 | 0.097 | 0.113 | 0.166 |
| ^12^C1, ^13^C2 | 0.033 | 0.016 | 0.053 | 0.173 | 0.190 | 0.128 | 0.261 | 0.214 | 0.288 |
| ^13^C1, ^13^C2 | 0.916 | 0.938 | 0.887 | 0.694 | 0.682 | 0.772 | 0.512 | 0.547 | 0.378 |
| **Formate** | | | | | | | | | |
| ^12^C | 0.836 | 0.917 | 0.851 | 0.526 | 0.529 | 0.593 | 0.576 | 0.445 | 0.473 |
| ^13^C | 0.164 | 0.083 | 0.149 | 0.474 | 0.471 | 0.407 | 0.424 | 0.555 | 0.527 |
| **Propionate - Methyl group** | | | | | | | | | |
| ^12^C | 0.912 | 0.958 | 0.898 | 0.775 | 0.749 | 0.817 | 0.698 | 0.692 | 0.677 |
| ^13^C | 0.088 | 0.042 | 0.102 | 0.225 | 0.251 | 0.183 | 0.302 | 0.308 | 0.323 |
| **Propionate - Methylene group** | | | | | | | | | |
| ^12^C | -- | -- | -- | 0.741 | -- | -- | 0.677 | 0.659 | 0.647 |
| ^13^C | NF | NF | NF | 0.259 | NF | NF | 0.323 | 0.341 | 0.354 |
| **Glycolate** | | | | | | | | | |
| ^12^C1, ^12^C2 | 0.107 | 0.118 | 0.147 | 0.128 | 0.195 | 0.155 | 0.255 | 0.209 | -- |
| ^13^C1, ^12^C2 | ND | ND | ND | ND | ND | ND | ND | ND | ND |
| ^13^C2 | 0.080 | ND | ND | 0.196 | 0.250 | 0.171 | 0.365 | 0.277 | ND |
| ^13^C1,^13^C2 | 0.813 | 0.882 | 0.853 | 0.676 | 0.555 | 0.674 | 0.380 | 0.514 | ND |
| **Succinate*** | | | | | | | | | |
| ^12^C | -- | -- | -- | 0.803 | -- | 0.772 | 0.659 | 0.695 | 0.690 |
| ^13^C | NF | NF | NF | 0.197 | NF | 0.228 | 0.341 | 0.305 | 0.310 |

* Pattern of the ^13^C satellites for succintate suggests that only the 2,3-^13^C_2_ isotopologue was significantly populated

NF = isotopologue model could not be fit, due to baseline distortions in DISPEL spectra

or unresolveable peaks

ND = ^13^C satellites not detected

**Table S8:** Results of HMM search for *fdhC* within all translated gene sequences within the long-read metagenome assembly (day 300). Only significant hits are shown (E-value < 1E3). The resulting bin / MAG for each positive gene / contig is shown, where applicable.

| **Gene ID** | **Corresponding contig** | **Bin Name** | **MAG Name** | **HMM E-value** |
| --- | --- | --- | --- | --- |
| contig_1647_3 | contig_1647 | Unbinned | NA | 5.50E-12 |
| contig_1944_6 | contig_1944 | polished_nanopore-bin.35 | Firmicutes_1 | 3.20E-56 |
| contig_2516_6 | contig_2516 | bin3.1 | Caldatribacteriota_2 | 9.40E-24 |
| contig_993_621 | contig_993 | bin4.2 | Methanothermobacter_2 | 3.00E-102 |
| contig_993_622 | contig_993 | bin4.2 | Methanothermobacter_2 | 3.40E-53 |
| contig_1107_22 | contig_1107 | bin14.1 | DTU068_1 | 4.60E-44 |
| contig_1107_23 | contig_1107 | bin14.1 | DTU068_1 | 1.60E-15 |
| contig_2005_34 | contig_2005 | Unbinned | NA | 4.50E-53 |
| contig_985_45 | contig_985 | polished_nanopore-bin.49 | Chloroflexota_1 | 8.40E-48 |
| contig_770_141 | contig_770 | bin12.1 | Acetomicrobium_1 | 3.00E-65 |
| contig_369_3 | contig_369 | Unbinned | NA | 2.70E-54 |
| contig_422_357 | contig_422 | Unbinned | NA | 5.80E-65 |
| contig_3178_3 | contig_3178 | Unbinned | NA | 9.40E-46 |

**Table S9:** Results of a BLASTp search against the NCBI nr database (May, 2023) of all positive *fdhC* hits detected with the HMM (Table S8). The results of the top hit are shown for each potential *fdhC* gene.

| **Gene ID** | **Bin Name** | **MAG Name** | **Description** | **Scientific Name** | **Query Cover** | **E value** | **Per. Ident** | **Acc. Len** | **Accession** |
| --- | --- | --- | --- | --- | --- | --- | --- | --- | --- |
| contig_1647_3 | Unbinned | NA | putative formate transporter 1 [Clostridium sp. C105KSO15] | Clostridium sp. C105KSO15 | 100% | 3E-144 | 99.0% | 208 | CUX61462.1 |
| contig_1944_6 | polished_nanopore-bin.35 | Firmicutes_1 | formate/nitrite transporter family protein [Clostridiales bacterium] | Clostridiales bacterium | 99% | 0 | 97.2% | 285 | NLZ91764.1 |
| contig_2516_6 | bin3.1 | Caldatribacteriota_2 | formate/nitrite transporter family protein [Candidatus Atribacteria bacterium] | Candidatus Atribacteria bacterium | 95% | 8E-86 | 99.2% | 258 | NLY05498.1 |
| contig_993_621 | bin4.2 | Methanothermobacter_2 | formate/nitrite transporter family protein [Methanothermobacter] | Methanothermobacter | 98% | 1E-106 | 100.0% | 274 | WP_074358763.1 |
| contig_993_622 | bin4.2 | Methanothermobacter_2 | putative formate transporter [Methanobacteriaceae archaeon 41_258] | Methanobacteriaceae archaeon 41_258 | 83% | 2E-46 | 98.8% | 274 | KUK01214.1 |
| contig_1107_22 | bin14.1 | DTU068_1 | formate/nitrite transporter family protein [Syntrophomonadaceae bacterium] | Syntrophomonadaceae bacterium | 99% | 6E-113 | 99.4% | 378 | NLH28058.1 |
| contig_1107_23 | bin14.1 | DTU068_1 | formate/nitrite transporter family protein [Syntrophomonadaceae bacterium] | Syntrophomonadaceae bacterium | 96% | 4E-103 | 100.0% | 378 | NLH28058.1 |
| contig_2005_34 | Unbinned | NA | formate/nitrite transporter family protein [Bacillota bacterium] | Bacillota bacterium | 100% | 0 | 99.6% | 260 | NLM65073.1 |
| contig_985_45 | polished_nanopore-bin.49 | Chloroflexota_1 | formate transporter FocA [Chloroflexota bacterium] | Chloroflexota bacterium | 94% | 0 | 100.0% | 287 | NLH06138.1 |
| contig_770_141 | bin12.1 | Acetomicrobium_1 | formate/nitrite transporter family protein [Acetomicrobium hydrogeniformans] | Acetomicrobium hydrogeniformans | 100% | 0 | 97.4% | 273 | WP_273001940.1 |
| contig_369_3 | Unbinned | NA | TPA: formate/nitrite transporter family protein [Syntrophaceticus sp.] | Syntrophaceticus sp. | 91% | 0 | 96.3% | 348 | HHY41208.1 |
| contig_422_357 | Unbinned | NA | TPA: formate transporter [Bacillota bacterium] | Bacillota bacterium | 100% | 0 | 99.6% | 272 | HCD41537.1 |
| contig_3178_3 | Unbinned | NA | formate/nitrite transporter family protein [Lachnospiraceae bacterium] | Lachnospiraceae bacterium | 100% | 0 | 97.3% | 302 | MBW4848334.1 |

**Table S10:** *fdhC* genes used to create the reference HMM that was used to query the long-read metagenome for potential unbinned *fdhC* genes (Tables S8 and S9).

| **Organism** | **Organism/Sequence GenBank/RefSeq accession number** | ***fdhC* locus tag** |
| --- | --- | --- |
| Methanobacteriaceae archaeon 41_258 | LGEU01000035.1 | KUK01214.1 |
| Methanobacterium bryantii M.o.H. | NZ_LMVM00000000.1 | LMVM01_120410 |
| Methanobacterium formicicum | CEA13820.1 | DSM1535_1487 |
| Methanobacterium formicicum Mb9 | CEL23790 | MB9_0134 |
| Methanobacterium paludis SWAN1 | CP002772.1 | MSWAN_2095 |
| Methanothermobacter | WP_048176094.1 | WP_048176094.1 |
| Methanothermobacter | WP_074358763.1 | WP_074358763.1 |
| Methanothermobacter defluvii | WP_115892643.1 | WP_115892643.1 |
| Methanothermobacter sp. CaT2 | NZ_AP011952.1 | BAM70688.1 |


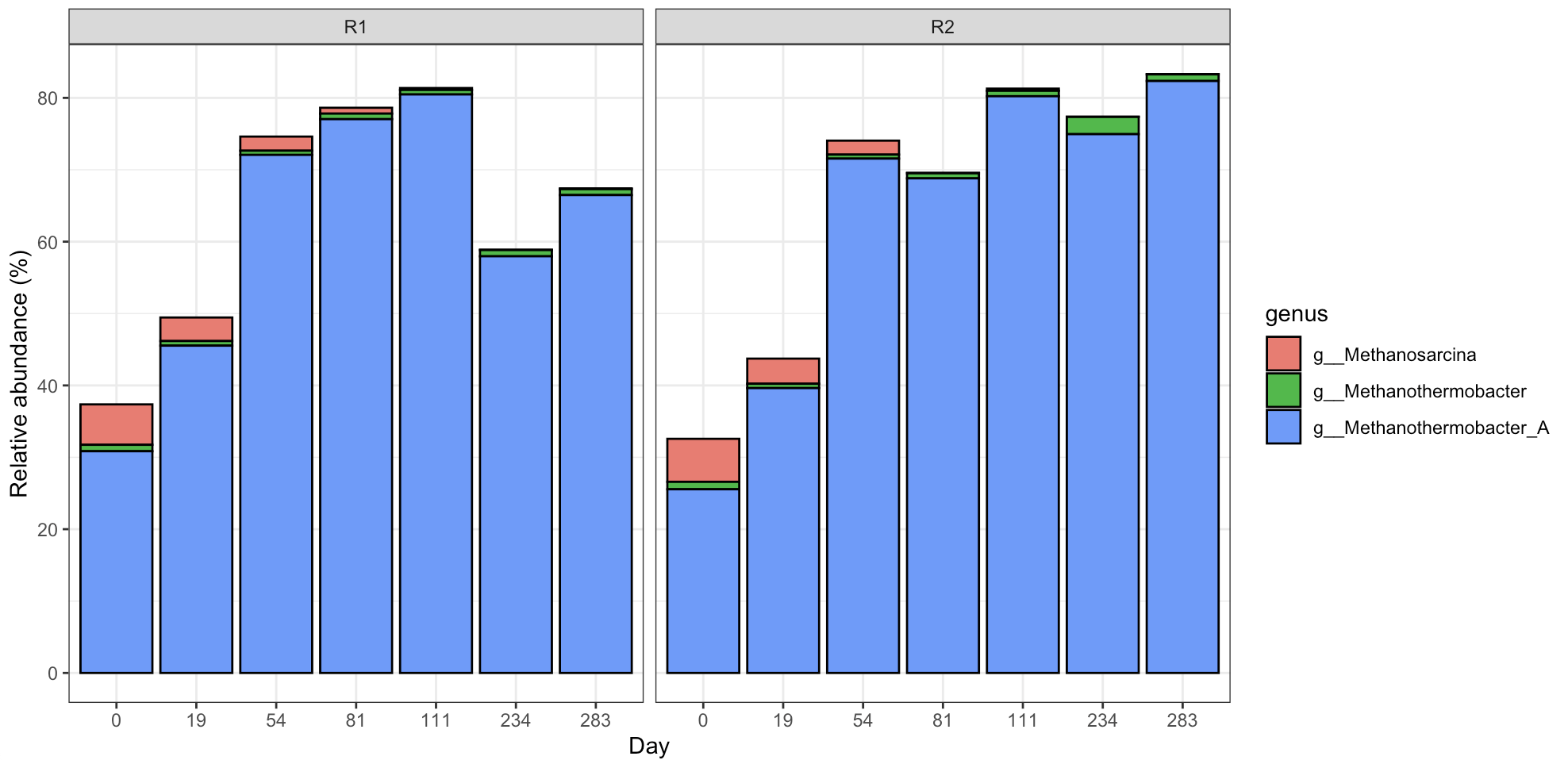


**Figure S1:** Abundance-weighted fraction (e.g. relative abundance) of archaeal genera in each bioreactor (R1 and R2), based on k-mer-based estimates of metagenome composition. K-mers (size = 31) were classified with sourmash using the GTDB v214, and were agglomerated at the genus level for visualization.

**Figure S2:** Heatmap of average nucleotide identity (ANI) values for reference *Methanothermobacter* genomes (NCBI accessions given in parenthesis), along with the *Methanothermobacter_1* and *Methanothermobacter_2* MAGs recovered in this study. ANI values were determined with FastANI.

**Figure S3:** Heatmap of average nucleotide identity (ANI) values for DTU068 genomes along with *Syntrophaceticus schinkii* within the same family of *Thermacetogeniaceae* (NCBI accessions given in parenthesis), along with the DTU068_1 and DTU068_2 MAGs recovered in this study. ANI values were determined with FastANI.


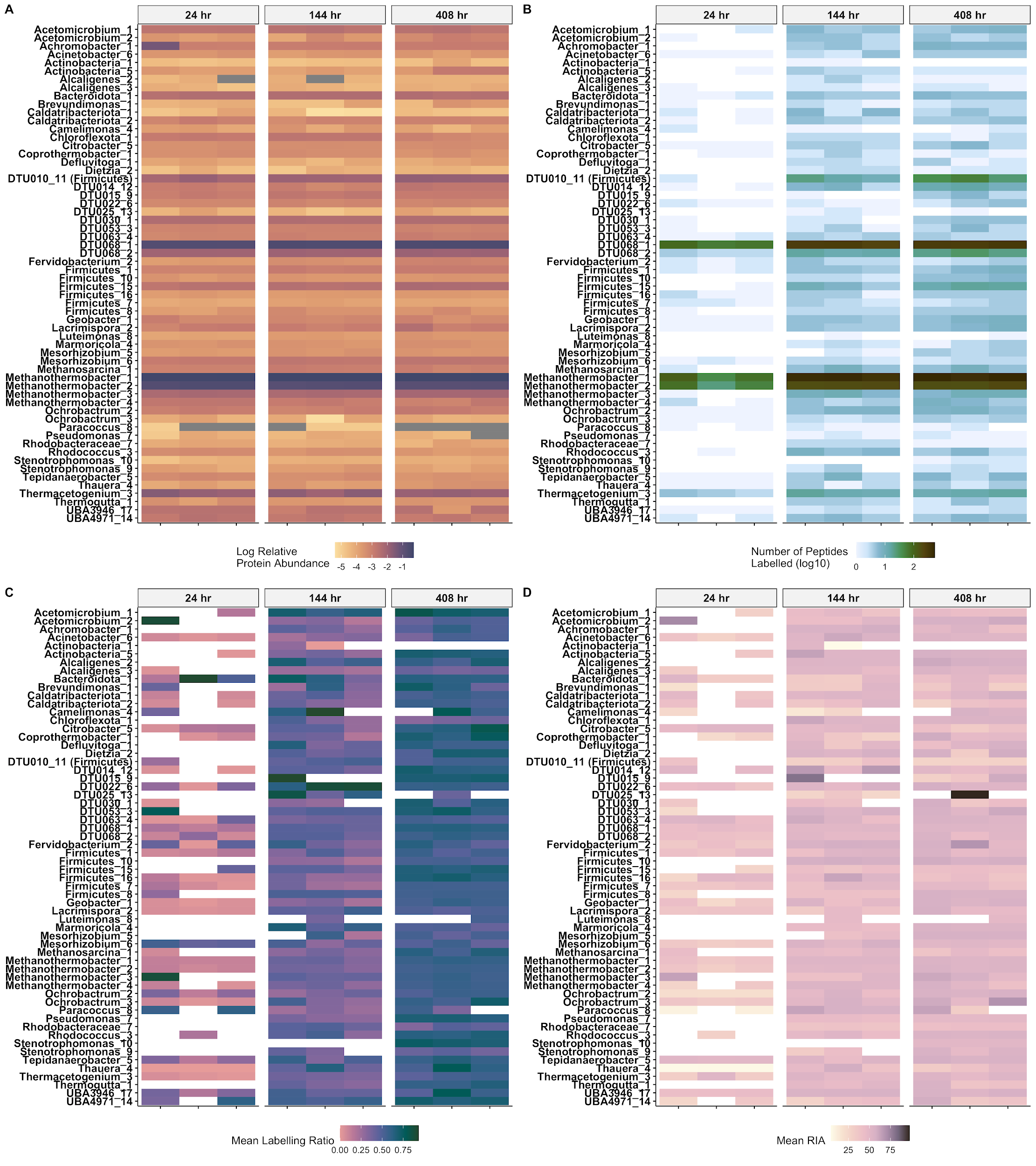


**Figure S4:** Heatmaps showing, for each of the 60 de-replicated MAGs recovered in this study at 24 hrs, 144 hrs, and 408 hrs of the SIP incubation: (A) relative protein abundance (log_10_-scaled, based on label-free quantification); (B) the number of ^13^C-labeled peptides identified; (C) the mean labeling ratio (LR) of ^13^C proteins within the genome; (D) the relative isotope abundance (RIA) of ^13^C proteins within the genome.

**Supplemental References:**

1. Federation WE, Association APH. Standard methods for the examination of water and wastewater. *Am Public Health Assoc APHA Wash DC USA* 2005.

2. Westerholm M, Moestedt J, Schnürer A. Biogas production through syntrophic acetate oxidation and deliberate operating strategies for improved digester performance. *Appl Energy* 2016; **179**: 124–135.

3. Kim S, Pevzner PA. MS-GF+ makes progress towards a universal database search tool for proteomics. *Nat Commun* 2014; **5**: 5277.

4. Käll L, Canterbury JD, Weston J, Noble WS, MacCoss MJ. Semi-supervised learning for peptide identification from shotgun proteomics datasets. *Nat Methods* 2007; **4**: 923–925.

5. Weisser H, Choudhary JS. Targeted Feature Detection for Data-Dependent Shotgun Proteomics. *J Proteome Res* 2017; **16**: 2964–2974.

6. Serang O, MacCoss MJ, Noble WS. Efficient Marginalization to Compute Protein Posterior Probabilities from Shotgun Mass Spectrometry Data. *J Proteome Res* 2010; **9**: 5346–5357.

7. Sachsenberg T, Herbst F-A, Taubert M, Kermer R, Jehmlich N, von Bergen M, et al. MetaProSIP: Automated Inference of Stable Isotope Incorporation Rates in Proteins for Functional Metaproteomics. *J Proteome Res* 2015; **14**: 619–627.

8. Søndergaard D, Pedersen CNS, Greening C. HydDB: A web tool for hydrogenase classification and analysis. *Sci Rep* 2016; **6**: 34212.

9. Losey NA, Poudel S, Boyd ES, McInerney MJ. The Beta Subunit of Non-bifurcating NADH-Dependent [FeFe]-Hydrogenases Differs From Those of Multimeric Electron-Bifurcating [FeFe]-Hydrogenases. *Front Microbiol* 2020; **11**.

10. Mulat DG, Ward AJ, Adamsen APS, Voigt NV, Nielsen JL, Feilberg A. Quantifying Contribution of Synthrophic Acetate Oxidation to Methane Production in Thermophilic Anaerobic Reactors by Membrane Inlet Mass Spectrometry. *Environ Sci Technol* 2014; **48**: 2505–2511.

9. Moutzouri, P.; Kiraly, P.; Phillips, A. R.; Coombes, S. R.; Nilsson, M.; Morris, G. A. C-13 Satellite-Free H-1 NMR Spectra. *Anal Chem* **2017**, *89* (22), 11898-11901. DOI: 10.1021/acs.analchem.7b03787.

10. Peat, G.; Kew, W.; Uhrin, D. 2D DISPEL-TOCSY: Using NMR to quantify 13C enrichment in metabolomics samples. Presented at the 2021 SMASH Small Molecule NMR Conference. Virtual Conference, 8/30/2021-9/2/2021. Poster. 2021.
